# Elucidating Surface Adsorption of Lithium Ions on Electrode Materials Using ^7^Li Dark-State Exchange Saturation Transfer NMR Spectroscopy

**DOI:** 10.1021/jacs.5c09603

**Published:** 2025-09-26

**Authors:** Shakked Schwartz, Ayan Maity, Vaishali Arunachalam, Yuval Bernard, Ortal Lidor-Shalev, Tehila Meshita, Liat Avram, Malachi Noked, Michal Leskes

**Affiliations:** † Department of Molecular Chemistry and Materials Science, 34976 Weizmann Institute of Science, Rehovot 7610000, Israel; ‡ Department of Chemistry, 108754 Bar -Ilan University, Ramat Gan 529002, Israel; § Bar -Ilan Institute of Nanotechnology and Advanced Materials, Ramat Gan 529002, Israel; ∥ Department of Chemical Research Support, 34976 Weizmann Institute of Science, Rehovot 7610000, Israel

## Abstract

Interfacial chemistry plays a central role in the development of next-generation high-energy Li-ion electrode materials. Yet, the rational design of new surface treatments that serve as beneficial solid electrolyte interphases is hindered by the challenges involved in probing their interfacial ion transfer properties. Here, we demonstrate how ^7^Li Dark-State Exchange Saturation Transfer (DEST) NMR can be used to directly measure the Li-ion surface adsorption process at the solid–liquid interface. The development of an optimized model system composed of monodisperse submicron particles allowed for comparison between different surface functionalities, enabling the characterization of state-of-the-art electrode coating layers in terms of their Li-ion affinity. Numerical simulations based on Bloch–McConnell equations enable a quantitative analysis of the surface exchange rates and binding properties, cementing DEST as a valuable tool for elucidating the structure–function relationship in electrode materials.

## Graphical abstract

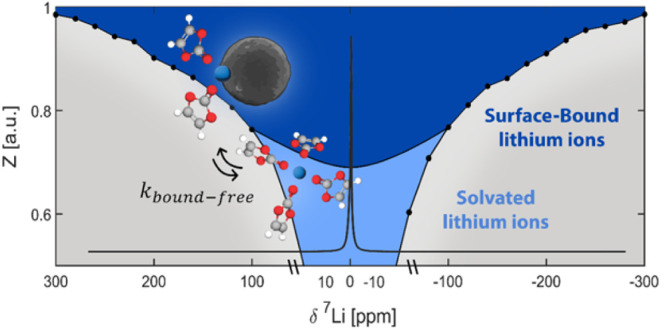

## 1 Introduction

The global shift in the energy sector toward electrification and renewable energy sources is contingent upon high-performing, efficient, and reliable rechargeable batteries. Lithium-ion (Li-ion) batteries, in particular, hold promise for the development of large-scale energy storage systems, which are highly demanding for electric transportation and grid-scale energy storage.^1^ Yet, implementing batteries in large-scale applications calls for developing materials with higher energy storage capabilities. This, in turn, requires understanding and achieving control over the underlying mechanisms driving capacity deterioration and cell failure in Li-ion batteries.^2^

Battery performance hinges on efficient Li-ion transfer through the different cell components and their interfaces. Of special significance is the electrode–electrolyte interphase (EEI), where the highly reactive electrode surface triggers decomposition of various electrolyte components, forming heterogeneous and disordered interfacial layers, namely the solid-electrolyte interphase (SEI) on the anode and the cathode-electrolyte interphase (CEI) on the cathode.^3–5^ The EEI properties are dictated by the electrolyte, as the oxidation and reduction potentials of its components, relative to the electrode redox potential, determine the decomposition products. The Li-ion solvation sheath also plays a role in engineering the EEI by bringing specific electrolyte components into close contact with the electrode, as well as shifting the HOMO–LUMO levels of the coordinated species.^6,7^ High ionic conductivity and stability of the EEI are essential for efficient cycling and capacity retention. As such, there is great interest in rationally designing EEIs by electrolyte engineering or the fabrication of artificial interphases to improve the overall performance and lifetime of the battery.^8^ As the EEI is situated at the solid–liquid interface, it plays a pivotal role in the multistep process of ion transfer from the electrolyte to the electrode: First, the solvent-encased Li ions must desolvate and preferentially bind to the surface.^9,10^ The Li ions must then diffuse through the various EEI phases toward the electrode, where they will finally intercalate or deposit, depending on the substrate electrode.^5,11,12^ Each of these steps is governed by a specific activation energy that is associated with the chemistry in different parts of the complex EEI. Specifically, the barrier for the first step depends on electrolyte desolvation and the binding of lithium ions by the outer layers of the EEI. The second and third steps depend on the EEI inner composition and the mechanism of ionic diffusion across the interphases.

Previously, several studies have focused on identifying which of these processes is rate determining. Following the seminal works by Ogumi et al. and Xu et al., where first step, of desolvation and binding, was found to have the highest energy barrier and was considered the bottleneck of cationic transfer process.^10,13–16^ Other studies found that migration through the EEI has a comparable barrier.^17,18^ Recently, it was also shown computationally that the energy barrier for the desolvation step can be lowered through EEI engineering, where functional groups on the surface can compete with the solvation sheath.^15^ It is reasonable to expect that the rate-determining step will vary in different electrolytes and in the presence of surface functionalities. However, as these interfacial ion-transfer processes are often measured in convolution, it is challenging to isolate their specific energy barriers and determine how they depend on electrolyte and EEI composition. Thus, to accurately portray this process, we must be able to detect these interfacial dynamics directly and separately. By doing so, we will be able to obtain a structure–function understanding of the surface chemistry—namely, determine which surface functionalities lead to too strong irreversible binding (that could result in making the first step limiting, as it would increase the energy barrier for further migration into the electrode and thus increase the overpotential of the cell) and which are too weak to compete with the electrolyte solvation shell. One can speculate that in analogy to heterogeneous catalysts, where the binding of the reaction intermediates to the catalyst should be “just right” following the Sabatier principle,^19,20^ similar behavior is likely for electrochemical interfaces. Clearly, the complexity of battery cells and the variability in lithium intercalation and deposition mechanisms suggest that the optimal binding strength may differ across materials and applications. Here, we aim to provide and implement the methodology that will offer an experimental route to derive such Sabatier relations for the EEI.

The chemistry and morphology of native and artificial EEIs have been studied extensively using various methods,^19–25^ however, elucidating the interfacial charge transfer properties of these interphases has proved to be far more challenging. Thus, despite being able to determine the chemical makeup of the surface layer, we have so far been unable to rationally predict or design a beneficial EEI for lithium transfer. Electrochemical impedance spectroscopy (EIS)^26–28^ is considered the established approach to determine the ionic resistivity of interfaces. However, with EIS it is impossible to extract information on a specific charge-transfer step from the multitude of processes that affect interfacial resistance.^13,29^ Similarly, isotope exchange measurements are useful for extracting a global exchange rate across the EEI,^30,31^ capturing a convolution of transfer processes without being sensitive to the separate steps. Additionally, these measurements are generally conducted on composite electrodes, which contain additives and binders mixed with the active materials, further complicating the results.^32^ Theoretical tools have also been used to address these questions,^12,33–35^ yet the complexity of modeling interfaces demands an experimental approach to individually characterize the different interfacial ion transfer processes and identifying the rate-limiting step.

Nuclear Magnetic Resonance (NMR) spectroscopy is a powerful tool for characterizing chemical environments as well as dynamic processes.^36,37^ However, the heterogeneity, anisotropy, and limited quantity of the EEI and the adsorbed Li ions render them invisible in the presence of the overpowering electrolyte signal. Removal of the electrolyte might reveal this environment but would also drastically change the surface properties. Probing the surface in its native wet form is crucial to gain a real understanding of its structure–function relationship.^38^ Previously, we demonstrated how Chemical Exchange Saturation Transfer (CEST) can be used to detect Li exchange between the EEI and Li metal in the presence of the liquid electrolyte.^39^ CEST reveals the charge-transfer step at the inner solid–solid interface, while the outer solid–liquid interface remains unexplored. Here, we employ a related approach, Dark-State Chemical Exchange Saturation Transfer (DEST),^40^ to shed light on the process of ionic adsorption on the EEI. Both CEST and DEST rely on saturation transfer for detecting the exchange process between an abundant visible population and a sparse hidden one; CEST is performed when the two environments have different chemical shifts (such as the Li in the metal and in the SEI^39^) while with DEST they overlap, which is often the case with solvated and surface-adsorbed species. Instead, the free and bound states are distinguished by their relaxation rate disparity, causing the NMR signal of the bound state to be broadened beyond detection due to its significantly faster transverse relaxation rate. In DEST, selective saturation of this hidden resonance is transferred to the visible free state via chemical exchange, enabling indirect detection of the bound state through the reduction of the free state. Evaluating the intensity reduction of the observed signal as a function of the saturation offset can be utilized to uncover the properties of the bound state and, most importantly, the exchange process. Formerly, saturation transfer experiments were developed in ^1^H NMR to probe exchange between small molecules and large aggregates in biological systems.^40,41^ More recently, the method was extended to study molecular adsorption onto the surface of nanoparticles suspended in a gel-like matrix.^39–42^

Here we first demonstrate how ^7^Li-DEST can be used to directly measure the Li-ion surface exchange process. To develop and optimize the method, we applied it in controlled model systems of monodispersed submicrometer TiO_2_ and SiO_2_ spherical particles. We then utilize the approach to compare the Li-ion adsorption capabilities of state-of-the-art surface coatings commonly employed to passivate the surfaces of electrode materials.^43–45^ Modeling of the DEST profiles enabled quantification of the surface exchange rates and the number of binding sites arising from different surface chemistries. Furthermore, we determine the surface composition with high sensitivity via dynamic nuclear polarization (DNP) surface-enhanced NMR spectroscopy (DNP-SENS).^49^ In DNP-SENS the high electron spin polarization of stable radicals is transferred to the surrounding nuclei by microwave irradiation, thereby increasing the surface NMR response. Coupling DEST with DNP-SENS allows us to sensitively observe and differentiate the surface species participating in the exchange and directly and accurately compare different electrode surfaces in terms of their Li-ion affinities.

With the presented ^7^Li-DEST approach, we are finally able to disentangle the elusive Li-ion interfacial processes, previously measured only in convolution, and characterize them in terms of their kinetics. We expect that this approach can be used to examine the role of specific surface species found in artificial as well as native EEIs, facilitating the design of efficient coatings with the desired properties and reverse engineering of electrolyte additives that would naturally create them. Moreover, this approach could be used to compare the desolvation energy of Li-ion solvation sheaths in different electrolytes. Utilizing this powerful tool on various battery systems could significantly expand our understanding of ion dynamics in batteries and pave the way for the design of new and improved electrolytes and interphases.

## 2 Materials and Methods

### 2.1 Particle Synthesis and Surface Coating

Monodisperse submicron TiO_2_ spheres were synthesized via hydrolysis and condensation of titanium isopropoxide (TIP, Sigma-Aldrich) catalyzed by *n*-octylamine and water in ethanol.^50^ Similarly, monodisperse submicrometer SiO_2_ spheres were synthesized via hydrolysis and condensation of tetraethyl orthosilicate (TEOS, Sigma-Aldrich), catalyzed by ammonium hydroxide and water in ethanol (Scheme 1). Control over the particle size was achieved by carefully adjusting the reaction time and temperature. Following synthesis, the powders were isolated by centrifugation, washed repeatedly with ethanol, dried overnight at 80 °C, and calcined at 550 °C for 6 h to obtain pure crystalline anatase TiO_2_ and amorphous SiO_2_. The submicron spheres were subsequently characterized using Scanning Electron Microscopy (SEM) to determine the particle size distribution and morphology.

**Scheme 1:**
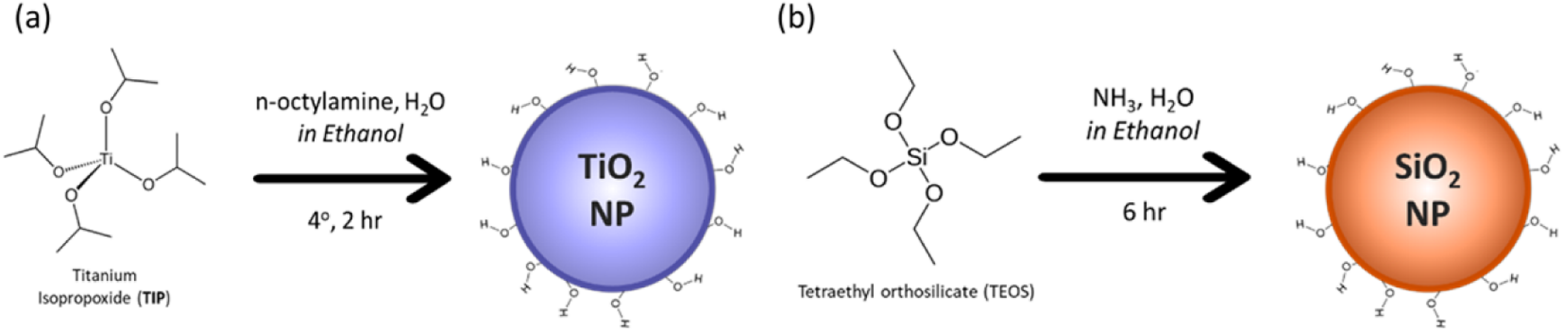
Overview of the Procedure for Synthesis of TiO_2_ and SiO_2_ Spheres

Coating of the TiO_2_ spheres in SiO_2_ was achieved by dispersing the particles in ethanol and repeating the SiO_2_ synthesis procedure with 1% wt of the TEOS precursor and a shorter calcination time. The uniformity and thickness of the SiO_2_ coating were confirmed using Transmission Electron Microscopy (TEM), while the surface chemistry was characterized using ^29^Si ssNMR spectroscopy.

Coating of the SiO_2_ spheres in Al_2_O_3_ was achieved via hydrolysis and condensation of 16 wt% aluminum isopropoxide (Sigma-Aldrich) catalyzed by water in toluene. Following the synthesis, the particles were washed repeatedly with ethanol, dried overnight at 80 °C, and calcined at 400 °C for 3 h. The uniformity and thickness of the Al_2_O_3_ coating were confirmed using TEM, while the surface chemistry was characterized using ^1^H and ^27^Al ssNMR.

Additionally, the SiO_2_ spheres were coated using molecular layer deposition (MLD) processes using an Ultratech Savannah 200 ALD. The particles were coated using a trimethylaluminum (TMA) precursor with two oxidizing agents: (i) Ethylene Glycol (EG) and (ii) Ethylenediamine (EDA). Prior to coating, 10 cycles of the O_3_ plasma treatment were performed to activate the surfaces. Additionally, this process was performed on the uncoated SiO_2_ spheres as a control experiment. 50 MLD cycles were performed to form the organic alumina coating variations with Ar as the carrier gas. The reactor temperatures were maintained at 135 °C for both procedures, while the EG and EDA cylinders were heated and stabilized at 95 °C. The MLD cycle consisted of a 0.1-s pulse of the organic precursor followed by a 15-s Ar purge, then a 0.2-s pulse of TMA followed by a 15 s Ar purge.

### 2.2 NMR Sample Preparation

#### 2.2.1 Solution NMR Spectroscopy

Following overnight drying in a vacuum oven at 100 °C, TiO_2_ and SiO_2_ powders were kept in an Ar-filled glovebox (O_2_, H_2_O < 0.5 ppm). To prepare the samples, an amount between 20 and 40 mg of each powder was weighed and packed in a 3-mm glass capillary. Liquid electrolyte was added to the powder, and the mixture was stirred within the capillary with a thin metal rod (<1 mm) to ensure complete and even wetting of the particles. The capillary was then placed in an airtight solution NMR tube. The optimal electrolyte:particle ratios were found to be 1:2 and 1:1 μL/μg for TiO_2_ and SiO_2_, respectively. LP30 electrolyte was used in almost all experiments performed here: 1 M LiPF_6_ in a 1:1 mixture of ethylene carbonate, EC, and dimethyl carbonate, DMC (Solvionic electrolyte grade, <20 ppm water), which will be referred to as the “LiPF_6_ electrolyte”. In one experiment aimed at testing the effect of free lithium content, the electrolyte was diluted to 0.2 M by the addition of 80 μL of a 1:1 mixture of anhydrous EC and DMC (Gotion) to 20 μL of the 1 M LiPF_6_ electrolyte.

#### 2.2.2 Solid-State NMR Spectroscopy

For solid-state Li-ion adsorption experiments, TiO_2_ and SiO_2_ powders were dried overnight in a vacuum oven at 100 °C and placed in an Ar-filled glovebox (O_2_, H_2_O < 0.5 ppm). The dried powders were soaked in LP30 electrolyte overnight, washed with anhydrous DMC to remove excess nonbound electrolyte salt and dried in the glovebox prechamber for 4 h. 10–20 mg of the powders were then packed into 4-mm zirconia rotors inside the argon glovebox.

For exogenous DNP-NMR measurements, TiO_2_ and SiO_2_ powders were dried overnight in a vacuum oven at 100 °C and placed in an Ar-filled glovebox (O_2_, H_2_O < 0.5 ppm). 15–25 mg of the powders were mixed with 10–15 μL of the radical solution, 16 mM TEKPol (Cortecnet) in tetrachloroethane (TCE, Sigma-Aldrich), resulting in slightly moist powders. The powders were packed into 3.2 mm sapphire rotors inside the argon glovebox. The rotors were closed with a Teflon plug and zirconia cap and inserted into the low-temperature probe, which was kept at about 100 K.

### 2.3 NMR Measurements

#### 2.3.1 Solution NMR Spectroscopy

All solution NMR measurements were performed on a 11.7 T Bruker AVANCE III spectrometer with 500.08 and 194.35 MHz Larmor frequencies for ^1^H and ^7^Li, respectively, using a 5-mm BBFO probe. All measurements were performed using Wilmad-LabGlass 5-mm Thin Wall Precision Vacuum NMR tubes. As the samples contained solid particles immersed in liquid, the inhomogeneity of the magnetic field was to be expected. Prior to each measurement, the magnet was shimmed by using an all-liquid sample, relating any residual inhomogeneity to the nature and organization of the particles in the sample. The pulse length was calibrated with respect to a flip angle of 90° and was equal to ∼11 μs and ∼15 μs for ^1^H and ^7^Li, respectively. The longitudinal and transverse relaxation rates of the lithium electrolyte were measured by using inversion recovery (IR) and Carr–Purcell–Meiboom–Gill (CPMG) experiments, respectively. The delays varied according to the sample conditions. ^7^Li and ^1^H spectra were acquired with a sufficiently long relaxation delay (steady state, 5 times the longitudinal relaxation time, *T*_1_) to ensure quantitative measurements.

DEST measurements were performed using the pulse sequence shown in the inset of Figure 1a, comprising a relaxation delay, continuous wave (cw) irradiation at varying off-resonance frequencies (w) to saturate the bound Li environment, followed by on-resonance acquisition (∼0 ppm) to detect the exchange with the visible environment. A recycle delay corresponding to 5T_1_ was used to ensure steady-state quantitative measurements. The saturation pulse ranged from 0.1 to 2 s in length, with an amplitude corresponding to nutation frequencies of 500–1500 Hz. The saturation pulse duration and amplitude of each measurement are specified in the different figure captions. Sample temperature and probe tuning remained stable over the entire saturation frequency range, which spanned from −500 to 500 ppm.

**Figure 1:**
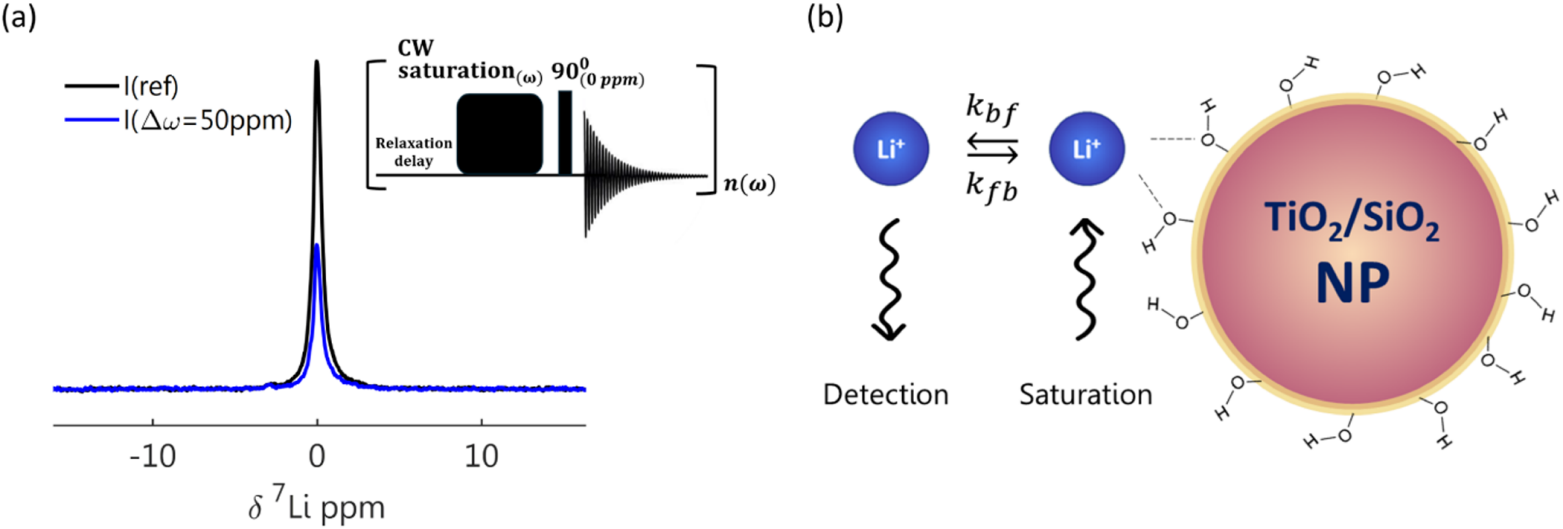
(a) ^7^Li NMR spectra of 1 M LiPF_6_ electrolyte in contact with solid particles obtained without and with presaturation labeled *I*_ref_ and *I* (△ω = 50 ppm), respectively. The inset illustrates the pulse sequence for the DEST experiment. (b) Illustration of the Li-ion exchange process occurring at the solid–liquid interface, where the hidden “surface-bound” lithium environment is saturated, followed by the surface exchange process, which partially transfers this saturation to the visible “solvated” lithium environment, resulting in a decrease in signal intensity.

All relaxation and DEST measurements were performed at 298, 313, and 323 K. The saturation pulse amplitude as well as the 90° acquisition pulse and recycle delay were calibrated at each temperature. Elementary spectral processing was done in Bruker TopSpin software, including phase and baseline corrections, followed by advanced processing and analysis in MATLAB (R2023B).

#### 2.3.2 Solid-State NMR Spectroscopy

Solid-state Li-ion adsorption NMR experiments were performed on a 9.4 T Avance-Neo spectrometer using a Bruker 4-mm triple resonance probe. ^7^Li spectra were referenced to LiF at −1 ppm. Samples were measured without sample spinning, with a sufficiently long relaxation delay to ensure quantitative measurements. The typical ^7^Li pulse length was 2.5 μs.

Exogenous DNP ssNMR experiments were performed on a Bruker 9.4 T Avance-Neo spectrometer equipped with a sweep coil and a 263 GHz gyrotron system. A 3.2-mm double-resonance low-temperature DNP probe was used for the experiments at magic-angle spinning of 9 kHz. All experiments were performed at about 100 K, with sample temperatures ranging between 100 and 110 K without and with microwave irradiation, respectively. All spectra were acquired after the sample temperature was stable. Longitudinal relaxation, *T*_1_, and polarization buildup time with microwave irradiation, *T*_bu_, were measured with the saturation recovery pulse sequence by using a train (50 repetitions) of short pulses separated by 1 ms delays for saturation. ^1^H spectra were acquired with a rotor-synchronized Hahn echo sequence using a 90 pulse of 3.125 μs. ^13^C and ^29^Si spectra were acquired using cross-polarization from ^1^H with nutation frequencies of approximately 51 for ^13^C and ^29^Si, and 60 kHz for ^1^H, during the spin lock period. ^27^Al spectra were acquired using the PRESTO sequence with ^1^H transfer.^51^ Relaxation measurements were analyzed using TopSpin and fitted with MATLAB (R2023B).

## 3 Results

### 3.1 ^7^Li DEST NMR for Measuring Lithium Binding

#### 3.1.1 Overview

To detect the Li-ion exchange at the solid–liquid interface, we have employed the DEST pulse sequence, which consists of off-resonance saturation and on-resonance detection (Figure 1). A long, low-power pulse is applied at a varying offset frequency (△ω) from the signal of the visible “solvated” lithium environment (defined as △ω = 0), saturating the “surface-bound” environment. Both environments are centered around △ω = 0, yet the “surface-bound” environment is too low in intensity to detect and/or broadened beyond detection with the experimental conditions optimized for the liquid signal. If there is no exchange between the solvated and surface-bound lithium ions, no reduction will be observed in the free Li-ion resonance (as the irradiation is off-resonance and applied with low power). If exchange between the two environments occurs at or faster than the time scale of the saturation, the visible signal, *I*(△ω) will exhibit partial saturation, resulting in the loss of signal. The ratio of signal lost compared to the unsaturated signal, I_ref_ is the measurable “DEST effect,” $Z\left(\Delta \omega \right)=\frac{I\left(\Delta \omega \right)}{I_{\mathrm{ref}}}$ (see Figure 1a).

Characterization of the hidden exchanging pool is achieved by performing the DEST experiment over a range of saturation offsets (△ω) and plotting the signal intensity as a function of those frequencies, resulting in the Z-spectrum or DEST profile, *Z*(△ω). As the saturation transfer occurs during the release of lithium ions from the surface, the exchange process we are sensitive to is between the bound and free Li ions with a rate given by k_bound‑free_, or *k*_bf_, in the equilibrium process: $p_{b}\times {\mathrm{Li}}_{\mathrm{free}}^{+}\overset{k_{fb}}{\underset{k_{bf}}{⇄}}{\mathrm{Li}}_{\mathrm{bound}}^{+}$ , with $p_{b}=\left[{\mathrm{Li}}_{\mathrm{bound}}^{+}\right]/\left[{\mathrm{Li}}_{\mathrm{free}}^{+}\right]$ describing the surface-bound Li-ion population. An increase in this exchange rate will result in broadening of the DEST profile as more saturated Li ions are released back to the electrolyte during the saturation time. Accordingly, a broad (or deep) DEST profile, as well as significant broadening with increasing temperature, indicates increased exchange, which corresponds to weak surface binding.

#### 3.1.2 Factors Affecting ^7^Li DEST Measurements

##### 3.1.2.1 Effect of Sample Preparation and NMR Parameters

The DEST measurement is sensitive to various sample conditions and measurement parameters, which can affect either the exchange rate *k*_bf_, the surface-bound Li-ion population *p*_b_, or the sensitivity of detection. To understand the optimal conditions and limitations of ^7^Li DEST for the detection of liquid-surface exchange, measurements were first conducted on model systems of monodisperse spherical TiO_2_ and SiO_2_ particles (Figure 2a,b). These perform well as model surfaces, as both can be produced with precise morphology and dimensions, which are crucial for differentiating the DEST contributions of the surface area and surface chemistry. Additionally, both particles are common building blocks for electrodes and electrode coatings in batteries,^52–55^ photocatalysis,^56,57^ and heterogeneous catalysis.^58–60^ Thus, understanding the chemical basis for the Li-ion surface dynamics revealed with DEST has significant value beyond the development of the approach, as it can provide insights into the surface properties of this important class of materials.

**Figure 2:**
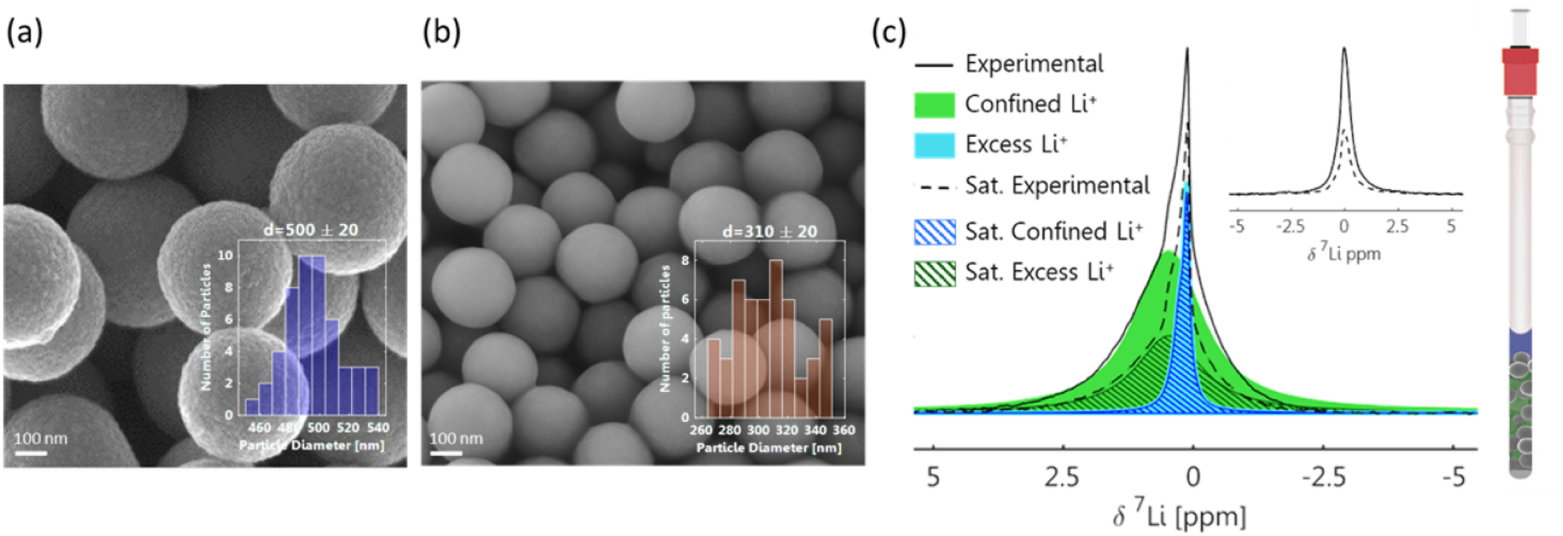
(a) SEM image of the TiO_2_ and (b) SiO_2_ spheres with average particle size of 500 ± 20 and 310 ± 20 nm, respectively. (c) ^7^Li NMR measurements conducted on TiO_2_ particles immersed in 1 M LiPF_6_ electrolyte. Deconvolution reveals two Li-ion signal components and their different contributions to *I*_ref_ (solid black) and *I* (△ω = 50 ppm) (dashed black) spectra obtained following a 2 s long saturation pulse with an amplitude of 1 kHz. While the confined Li^+^ resonance is affected by saturation (full to hatched green), the excess Li^+^ resonance remains unchanged upon saturation (overlapping full to hatched blue). The inset displays the ^7^Li NMR spectrum, before and after saturation, of the optimized sample, now containing only the confined Li^+^ resonance.

To assess the optimal sample conditions for the detection of liquid-surface exchange using DEST, ^7^Li NMR measurements were conducted on TiO_2_ particles immersed in a lithium electrolyte. Careful analysis of the ^7^Li spectrum (Figure 2c) revealed the contribution of two environments. These were deconvoluted to broad and narrow resonances, corresponding to the electrolyte surrounding the TiO_2_ particles (labeled confined Li^+^) and to the excess electrolyte, respectively. Comparing the spectra with and without low power saturation at 50 ppm confirmed that only the portion of the electrolyte in direct contact with the particles was affected by off-resonance saturation, indicating its participation in the surface exchange process. To maximize *p*_b_ (i.e., the fraction of the Li-ion population that participates in the exchange process), the electrolyte volume was fine-tuned to cover the powder precisely without any excess, resulting in an electrolyte-to-powder ratio of 1:2 μL/μg. The ^7^Li spectrum (inset of Figure 2c) of a sample prepared without the excess electrolyte indeed shows only the confined Li resonance, which is sensitive to saturation. Following optimization of the sample, a full DEST profile was acquired at 298 and 323 K (Figure 3a). Typically, the DEST profile is composed of two contributions: (i) direct saturation of the electrolyte confined within the interparticle porosity, which leads to limited broadening following irradiation close to 0 ppm, and (ii) saturation transfer due to exchange, which leads to a DEST effect over a broader region of frequencies. The width of the direct saturation region depends on the transverse relaxation of the confined electrolyte, while the width further from 0 ppm provides insights into the effect of surface exchange and the properties of the bound environment. As can be appreciated from Figure 3a, a significant DEST effect is observed—indicating the presence of substantial exchange between the broad resonance of the bound state and the narrow resonance of the free electrolyte centered at the same chemical shift. To fully appreciate the DEST measurement’s depiction of the surface exchange, a reference DEST profile acquired on a pure electrolyte sample is also plotted, displaying the breadth disparity (as well as highlighting the change in electrolyte properties once it is confined within the particles, resulting in a broader direct saturation effect).

**Figure 3:**
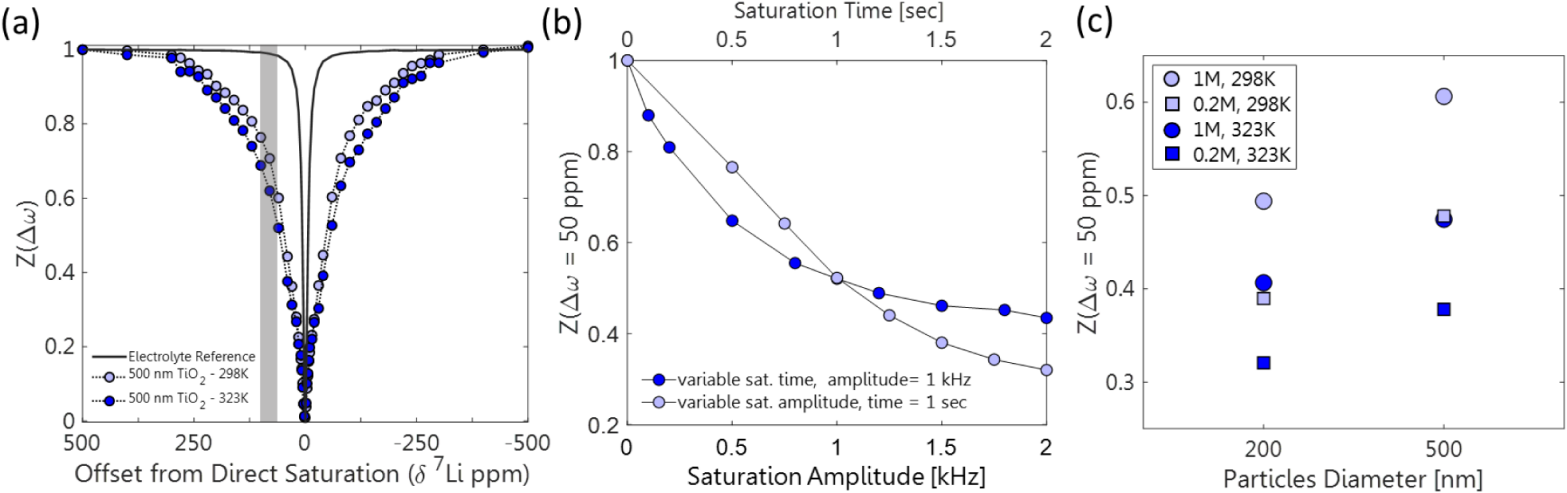
(a) DEST profile of 500 nm TiO_2_ particles immersed in 1 M LiPF_6_ electrolyte at 298 and 323 K, obtained with 1 kHz and 1 s saturation pulse. The signal was normalized to the spectrum obtained with saturation at 500 ppm and compared with a pure electrolyte DEST profile. (b) The DEST value, *Z* (△ω = 50 ppm) illustrated by the gray line in (a), plotted as a function of the saturation pulse amplitude (with fixed saturation time) and duration (with fixed saturation amplitude). (c) The DEST effect obtained with saturation at 50 ppm on the 500 nm and 200 nm TiO_2_ particles, measured at electrolyte concentrations of 1 and 0.2 M at 298 and 323 K. Measurements were performed using 1 kHz and 1 s saturation pulses.

To evaluate the dependence of the DEST profile on the saturation amplitude and electrolyte concentration, we performed DEST measurements with a fixed saturation offset of 50 ppm, where a significant DEST effect can be observed for the confined electrolyte. The effect of saturation amplitude and duration was evaluated (Figure 3b) and as expected, the DEST effect increased with increasing saturation power and duration, plateauing at about 1500 Hz saturation amplitude and 1.5 s saturation time. In practice, the choice of the saturation pulse was based on these results as well as technical considerations, which place a constraint on the duty cycle of the NMR probe. The DEST effect is also influenced by the available surface area of the particles and the electrolyte concentration, both controlling the exchanging fraction of Li-ion population p_b_. Decreasing the particle size and the electrolyte concentration should increase the available surface area (for fixed sample weight) and the exchanging fraction, respectively, thereby increasing the measured DEST effect. This increase was observed in both the variable concentration and particle size measurements. The difference in DEST effect between measurements of TiO_2_ particles with varying sizes of 500 ± 20 and 200 ± 30 nm was comparable to the difference in the geometrical surface area, considering also the effect of particle packing (see Sections S1.1–S1.2). Increasing the surface-to-volume ratio of the particles (and thus *p*_b_) by a factor of 2.4 ± 0.4 resulted in an increased DEST effect by a factor of 2 ± 0.5 (Figure 3c). Decreasing the electrolyte concentration also results in an increased DEST effect due to the increase in *p*_b_. Finally, with an improved understanding of the optimal sample and saturation conditions, we also examined the measurement sensitivity toward changes in k_bf_ through variable temperature measurements. We found that higher temperatures produced a slight increase in the DEST effect, indicating an increase in the exchange rate, in agreement with the Arrhenius law (Figure 3a).

##### 3.1.2.2 Effect of Surface Chemistry

Following the optimization and validation of the method, the surface chemistry dependence of the Li-ion exchange process was studied in the TiO_2_ and SiO_2_ particles. The ^7^Li DEST effect was evaluated before and after the deposition of a SiO_2_ coating layer on the TiO_2_ particle surface, as well as on bare SiO_2_ particles. The particles were initially characterized by TEM (Figure 4a), which clearly showed the deposition of a 15-nm-thick SiO_2_ layer on the TiO_2_ particles. In-depth chemical analysis of the outer surface layers was obtained by ^29^Si DNP-SENS,^61^ which was used to enhance the NMR signal in order to accurately detect and determine the external surface species that are in contact with the electrolyte. Employing ^1^H–^29^Si cross-polarization (CP) provided a significant increase in sensitivity. This measurement is biased toward the exposed surface species and against the proton-deficient particle bulk, and thus provides selectivity in the detection of the outer surface layers that are relevant for the surface adsorption process. The surface of both the SiO_2_ particles and the SiO_2_-coated particles were found to contain silanols (SiO)_3_SiOH as well as siloxane bridges (SiO)_4_Si, commonly known as Q3 and Q4 sites, in different proportions (Figure 4b).^62^ With deconvolution of the ^29^Si signal, the ratio of (SiO)_3_SiOH to (SiO)_4_Si was found to be larger on the surface of the SiO_2_ particles compared to the SiO_2_ coating, amounting to 2:1 and 1.5:1, respectively.

**Figure 4:**
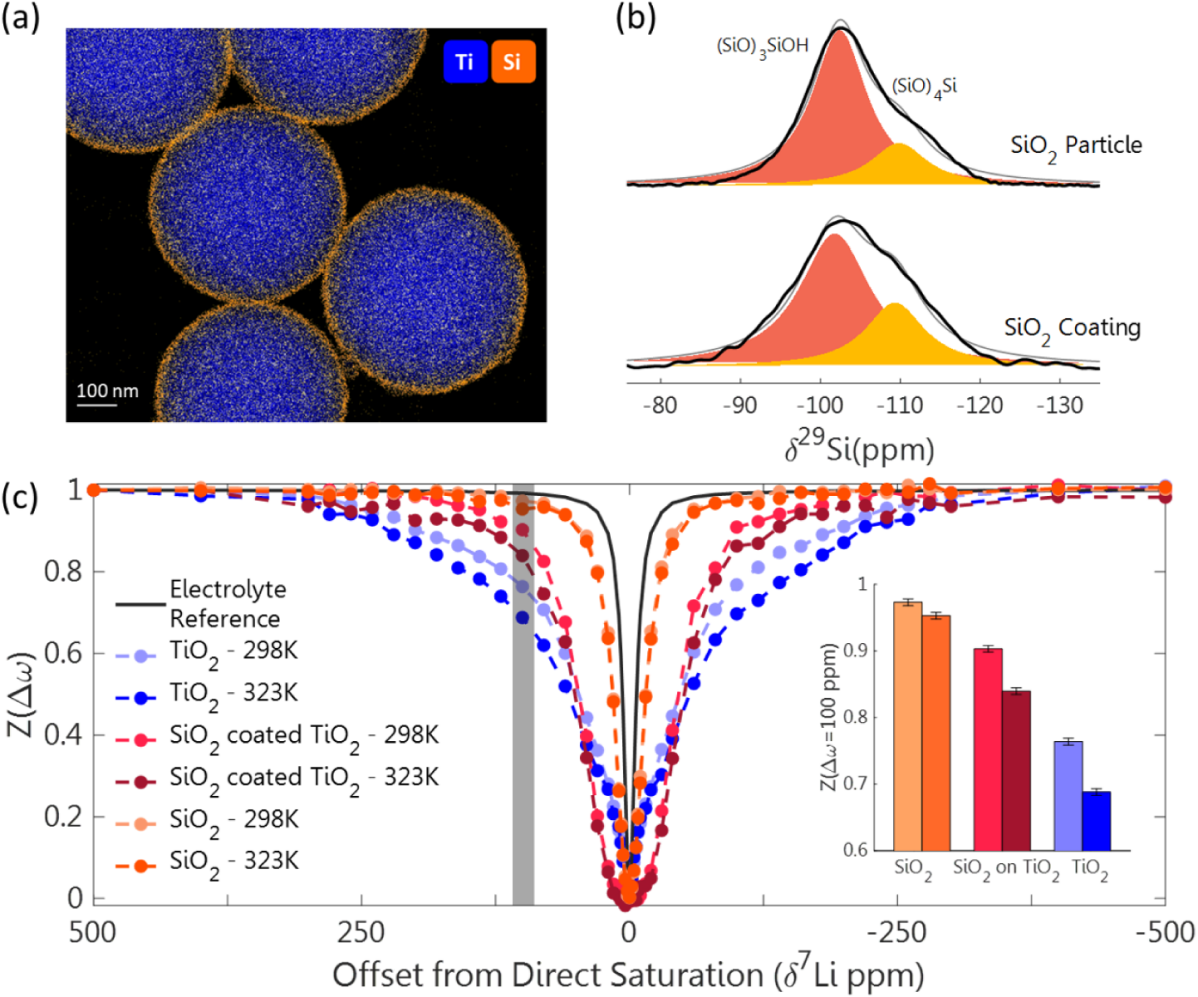
(a) TEM-EDX image of the ∼15-nm-thick SiO_2_ coating layer on the TiO_2_ particles. (b) ^29^Si ssNMR DNP-SENS spectra of SiO_2_ particles and a SiO_2_ coating layer acquired through ^1^H–^29^Si CP with a contact time of 4 ms and a spinning rate of 9 kHz. Deconvolution of the peaks reveals the ratio of silanols (SiO)_3_SiOH (orange) and siloxane bridges (SiO)_4_Si (yellow). (c) ^7^Li DEST profiles of the TiO_2_, SiO_2_, and SiO_2_-coated TiO_2_ particles immersed in 1 M LiPF_6_ electrolyte at 298 and 323 K, measured with a 1 kHz and 1 s saturation pulse, compared with a pure electrolyte DEST profile (black). The inset displays the ^7^Li DEST effect following saturation at △ω = 100 ppm, illustrated by the gray line, for the same systems and temperatures. Error bars were calculated based on the signal-to-noise ratio of the ^7^Li resonance.

Following immersion in the Li-ion electrolyte, the three surface types displayed significantly different ^7^Li-DEST profiles (Figure 4c), demonstrating the sensitivity of the method to various surface exchange regimes. The largest DEST effect is observed for TiO_2_ particles. Significant narrowing of the DEST profile is observed when going from the TiO_2_ to the SiO_2_ systems, with the SiO_2_-coated TiO_2_ displaying an intermediate exchange profile. The inset in Figure 4c displays the DEST effect for each surface and temperature at a fixed saturation offset of 100 ppm, where a significant DEST effect (signal reduction) can be observed, highlighting the differences between the surface chemistries. Considering first the SiO_2_ particles, we observe that they display no DEST effect at all. This could be a result of either practically irreversible binding of Li ions to the silica surface (with no or extremely slow exchange with the free electrolyte) or a negligible number of surface-binding sites for lithium ions. A narrow DEST effect can also be observed in the case of a bound state with very long transverse relaxation (as we will discuss in the following sections), yet this option is unlikely based on solid-state NMR measurements performed on SiO_2_ particles that were soaked in electrolyte (see Section S1.3 and Figure S3). To test whether the narrow profile is due to the limited number of binding sites, we have also acquired a DEST profile from dendritic fibrous nanosilica (DFNS),^63^ particles that have approximately 10 times higher surface area, which also showed no appreciable DEST profile (see Section S1.3 and Figure S2). Finally, we have also performed the DEST experiments at 50 °C, which again showed no DEST effect in the case of silica but an appreciable broadening for titania, indicating an increase in the exchange rate. Thus, we conclude that indeed the cause for no measurable DEST effect in SiO_2_ is the strong binding of ions to the surface.

The silica coating shows a slightly broader DEST profile, mostly in the spectral region of direct saturation but also at larger saturation offsets. Comparing the relaxation properties of the free electrolyte confined within the different particles, we indeed observed more than 1 order of magnitude decrease in the transverse relaxation time of the free electrolyte within the silica-coated TiO_2_ particles (1 ms), compared to that measured with uncoated titania and silica (distributed between 0.1 and 1 s). This could explain the broadening in the DEST profile of the silica coating around 0 ppm. Furthermore, at higher temperatures, the DEST effect increases for the silica-coated particles in contrast with bulk silica (though not to the extent observed for TiO_2_). Thus, our DEST and surface-sensitive NMR results suggest that bulk and thin-film SiO_2_ possess different surface properties, leading to the observed growth in the DEST effect, which is a function of both k_bf_ and p_b_. The stronger Li binding to silica particles compared to that of the silica coating might be due to the increased hydroxyl content found in DNP-SENS. In this case, lithium-proton exchange may result in strong surface affinity, a topic we will explore in future work. Importantly, these results highlight the sensitivity of the DEST approach to minute changes in surface properties and its suitability as a tool to probe surface exchange processes to fine-tune lithium binding. To examine this, we employ an approach to study different surface functionalities obtained via synthetic surface modifications.

### 3.2 Binding Affinity of Different Surface Functionalities

The now optimized ^7^Li DEST method was employed to examine the binding affinity of different surface treatments commonly used as protective coatings for electrodes.^46–48^ First, the SiO_2_ particles were coated with a thin film of Al_2_O_3_, which is widely used for surface passivation of anodes and cathodes.^64–66^ The coating was characterized by TEM (Figure 5a), which confirmed the presence of a uniform coating on the silica particles with a thickness of 15 nm. ^27^Al ssNMR (Figure 5b), enabled by DNP-SENS, revealed that the thin coating contains mostly 5- and 6-coordinated aluminate species and, to a lesser extent, of 4-coordinated Al environments.^67^

**Figure 5:**
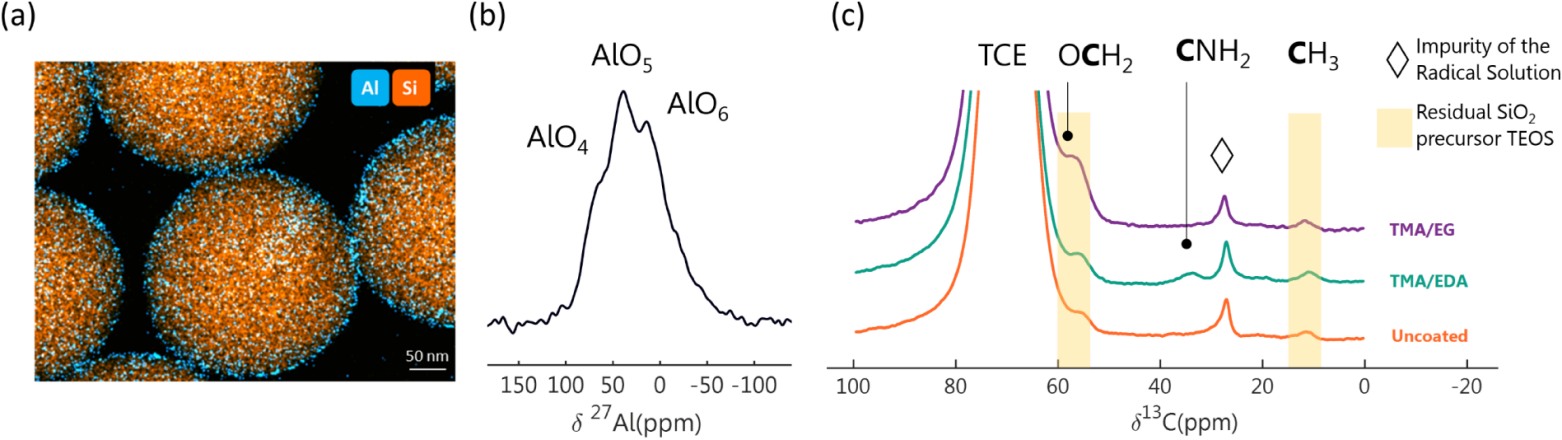
(a) TEM-EDX image of the Al_2_O_3_-coated SiO_2_ particles. (b) ^27^Al ssNMR spectrum of the Al_2_O_3_-coated SiO_2_ particles, enabled by DNP-SENS with ^1^H–^27^Al PRESTO used for polarization transfer of 1 rotor cycle, acquired with 3072 scans and a spinning rate of 9 kHz. (c) DNP-enhanced ^13^C ssNMR of the organic-aluminum hybrid coatings compared to the uncoated SiO_2_ particles. Spectra were acquired through ^1^H–^13^C CP with a contact time of 4 ms and a spinning rate of 9 kHz.

^7^Li DEST was measured on Al_2_O_3_-coated SiO_2_ particles in a Li-ion electrolyte as previously described. Z-spectra were acquired at five power levels and three temperatures (298, 313, and 323 K; Figure 6). The profiles showed significant broadening and strong temperature dependence. This points to Al_2_O_3_ being a weakly coordinating surface, which readily releases Li ions. In an attempt to improve the affinity of the Al_2_O_3_ coating to Li ions, the SiO_2_ surface was coated with organically modified aluminum hybrids known to stabilize and improve the performance of Li-ion battery electrodes.^46,68^ These hybrids were formed by Molecular Layer Deposition (MLD) using an alternating sequence of exposure to trimethylaluminum (TMA) and oxidizing precursors, Ethylene Glycol (EG) and ethylenediamine (EDA). Each coating was formed through 50 cycles of the MLD processes. DNP-SENS ^1^H–^13^C cross-polarization was used to detect and differentiate the minute quantity of surface species, as ssNMR alone is not sensitive enough to detect them. This enabled accurate determination of the outer surface layer, which is crucial for understanding the functional groups that will undergo Li^+^ exchange with the electrolyte. The TMA-EG and TMA-EDA coatings displayed resonances corresponding to alkoxy and amine surface groups, respectively, confirming the presence of the expected surface species based on the precursors used in the MLD process (Figure 5c). In both coated and uncoated SiO_2_ samples, resonances of methyl and alkoxy groups can be observed at 12 and 55 ppm, respectively, which we have determined to originate from partially decomposed TEOS precursor trapped inside the bulk of the particle (Section S1.4 and Figure S5).

**Figure 6:**
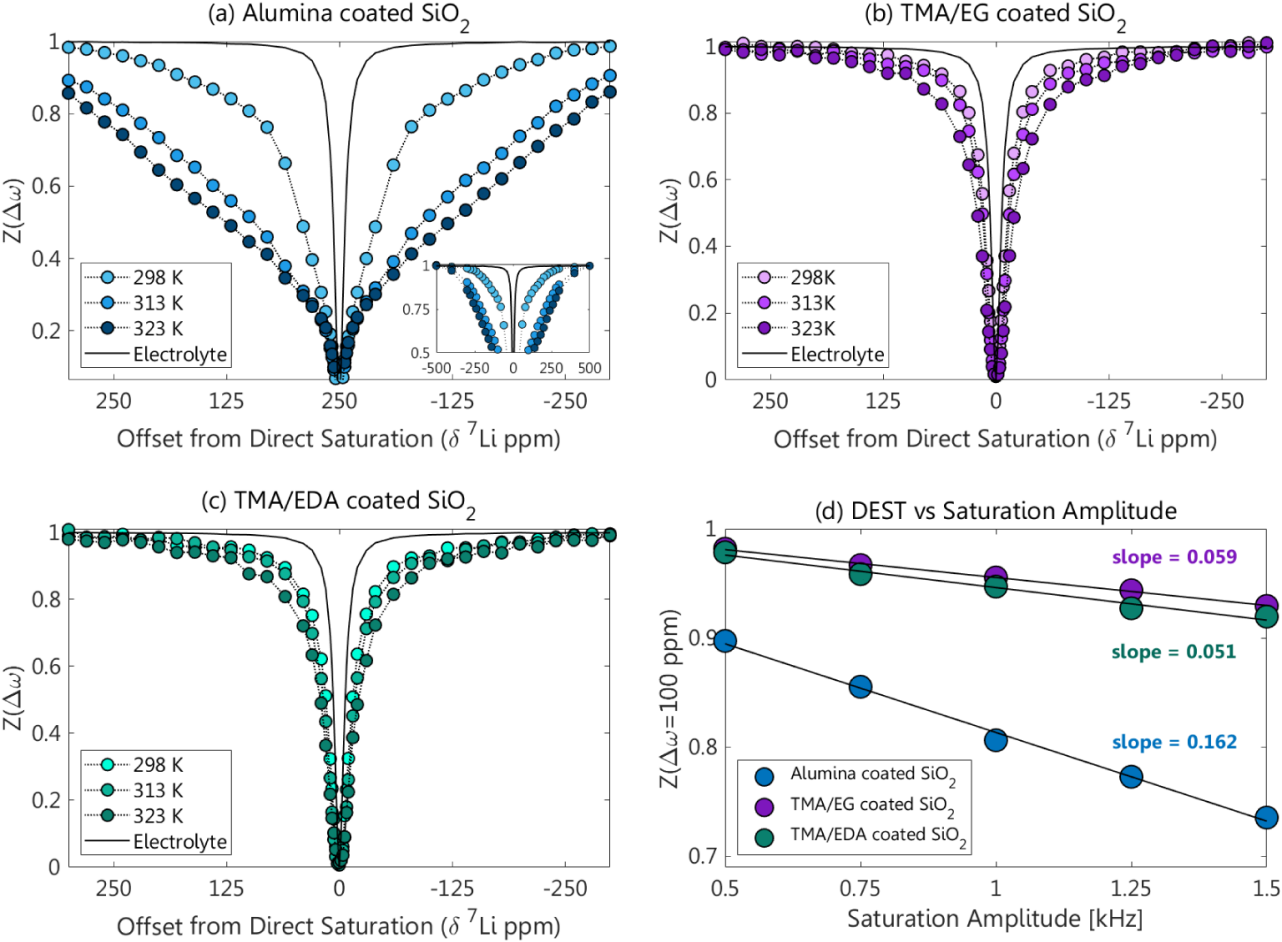
^7^Li DEST profiles of the (a) Al_2_O_3_ and (b, c) organic-aluminum hybrid-coated SiO_2_ systems measured at 298, 313, and 323 K. The profiles were measured using 1 kHz and 1 s saturation pulses, normalized to the spectrum obtained with a 500 ppm saturation offset, and compared to a pure electrolyte DEST profile (black). (d) Displays the variation in DEST response for each surface at 298 K with saturation at 100 ppm and the slope $\frac{\partial \left(Z\left(\Delta \omega =100\;\mathrm{ppm}\right)\right)}{\partial \left(\mathrm{Saturation}\;\mathrm{Amplitude}[\mathrm{kHz}]\right)}$ .

Samples of the TMA-EG- and TMA-EDA-coated SiO_2_ were prepared and characterized by ^7^Li-DEST similarly to those of the Al_2_O_3_-coated SiO_2_. The organic functionalization of aluminum coatings produced significantly narrower DEST profiles compared to the Al_2_O_3_ coating with much milder broadening as the samples were heated (Figure 6a–c), indicating increased Li-ion surface adsorption. As previously observed, the DEST effect grows with increasing temperature due to the more rapid release of Li ions from the surface. The stark difference between Al_2_O_3_ and the organic-aluminum coatings suggests that the addition of polar organic groups improves Li-ion surface binding. This effect may be due to improved Li-ion affinity of the binding sites, which would result in slower *k*_bf_. Conversely, the broadening observed for the Al_2_O_3_ coating could be due to an increased density of binding sites, which would increase *p*_b_.

It was shown that the acquisition of saturation profiles at variable saturation power can be used to disentangle the contributions from *k*_bf_ and *p*_b_, which is commonly employed for CEST based on analytical derivations,^69,70^ as well as for DEST in a more qualitative manner.^41,71^ Typically, with higher p_b_, which in our case indicates a larger number of surface binding sites, one expects a stronger dependence of the DEST response on the saturation power compared to the effect of the exchange rate (Figure S9). Here, we qualitatively evaluate this dependence by comparing the slope $\frac{\partial \left(Z\left(\Delta \omega =\mathrm{100 ppm}\right)\right)}{\partial \left(\mathrm{Saturation Amplitude}[\mathrm{kHz}]\right)}$ obtained for the different surfaces. The much larger slope observed for the alumina coating (0.16 vs 0.05–0.06, respectively, as shown in Figure 6d) compared to the organic functionalization suggests that this effect is due to a change in *p*_b_. Thus, possibly, the introduction of organic moieties reduces the number of surface binding sites. Yet the milder temperature dependence of the DEST effect suggests that *k*_bf_ remains slow throughout the measured temperature range, indicating that the binding sites of the organic-aluminum coatings have greater Li-ion affinity. In order to determine the binding affinity of different chemistries in a more quantitative way, we now turn to modeling the DEST profiles.

#### 3.2.1 Quantitative Analysis of the DEST Profiles

So far, we have conducted a qualitative comparison of the Li-ion binding properties of various surface chemistries using DEST. A quantitative analysis requires an in-depth understanding of the different parameters affecting the measurement and the resulting DEST profile. The surface exchange rate can be extracted from the experimental profiles by fitting the data to a two-pool exchange model based on Bloch–McConnell (BM) differential equations, which describe the evolution of magnetization undergoing saturation and relaxation as well as the exchange process (see Section S1.6).^72^ The model comprises a free Li-ion spin population that is in exchange with a surface-bound spin population. The relative populations are defined through the exchange rates: $p_{b}=\frac{k_{\mathrm{free}-\mathrm{bound}}}{k_{\mathrm{bound‐free}}}=\frac{k_{fb}}{k_{bf}}$ . Furthermore, the two spin populations are described by their longitudinal and transverse relaxation rates, as well as their chemical shifts. The analytical solution to the BM set of equations employs a simplified model valid under certain assumptions regarding the relaxation and exchange rates, which are not necessarily met in our system.^72^ Instead, we utilized the numerical approach, which operates by iteratively estimating the solution and testing its accuracy until the best solution is obtained, as evaluated by least-squares fitting to the data.^73,74^

Due to the large parameter range and to ensure accurate and reliable fittings, we performed additional experiments as well as extensive numerical simulations to establish realistic boundary conditions for our experimental systems. Supporting experiments were conducted to pinpoint realistic ranges for p_b_ and relaxation rates for $Li_{\mathrm{free}}^{+}$ (taking into account the effect of confinement^75^) and $Li_{\mathrm{bound}}^{+}$ (see Section S1.5). Moreover, numerical simulations of the Z-spectra using the BM exchange model with a plausible range of parameters were conducted for the investigated systems (Figure S8). The resemblance of the simulations to our experimental profiles suggests that the proposed parameters are realistic for our systems.

With this understanding of the relevant range of parameters, we turned to numerically fit our experimental DEST results. The results obtained for the Al_2_O_3_ and organic-aluminum hybrid coatings at 298 K were fitted using the numerical solution to the two-pool BM exchange model (Figure 7). Interestingly, we found that the Al_2_O_3_ coating (formed through solution processing as well as a sample coated via atomic layer deposition, which is not shown here) displayed surface reactivity at higher temperatures (Figure S6), instigating changes to the surface binding sites during the DEST measurements. This is in agreement with previous studies in which Al_2_O_3_ cathode coatings were found to react with LiPF_6_ carbonate electrolytes, thereby changing the chemistry of the coating.^76–78^ Understanding these chemical changes and their effect on the Li-ion surface adsorption dynamics is the subject of an ongoing investigation. Accordingly, the results obtained for the Al_2_O_3_ coating were fitted only at room temperature (where the surface remained unreacted, Figure S6), while the results obtained for the organic-aluminum hybrid coatings were fitted at all three temperatures and compared with the DEST response of TiO_2_ particles that served as a reference for an unreactive inorganic surface (Figure S10). The data sets for each temperature were fitted independently using the multiple Z-spectra acquired with varying saturation amplitude as input. The fitting parameters and boundaries for each data set are detailed in Table 1. The longitudinal relaxation rate and chemical shift of the free Li ions, *R*_1f_ and △*ω*_f_, respectively, were fixed to their experimentally determined values under the assumption of negligible confinement effects. The longitudinal relaxation rate of the bound species was fixed at 0.1 Hz as it did not vary much between data sets, and its fitted value matched the ^7^Li ssNMR measurement of the dry Li-adsorbed surface (Figure S7). The initial value and upper boundary condition of the transverse relaxation rate of the free Li ions R_2f_ were adjusted based on multiple fitting attempts. Thus, only four unknown parameters remained to be fitted: k_bf_, *p*_b_, R_2f_ and R_2b_ (the bound-to-free exchange rate, the bound population, and the free and bound transverse relaxation rates). Furthermore, as the population and exchange rate of the bound species are somewhat interchangeable (both can induce broadening of the DEST profile), we opted to fix the bound population fraction *p*_b_ for each surface chemistry in order to clearly observe the effect through the exchange rate alone. For the TiO_2_, Al_2_O_3_, and organic-aluminum hybrid coatings, *p*_b_ was fixed at 0.028, 0.03, and 0.002, respectively, following multiple fitting attempts which independently arrived at these values, within errors that were consistently an order of magnitude smaller. This large difference between the fitted population values obtained for the inorganic vs organic interphases is reasonable, considering the bulkiness of the organic moieties. As the coatings do not contain any innate lithium species, magnetization transfer can be excluded,^79^ as well as any significant spin–spin interactions that could affect the relaxation rates. As such, the magnetization parameters of all DEST profiles should fall within the same boundaries, and the DEST effect can be directly correlated with the Li-ion surface exchange rate.

**Figure 7:**
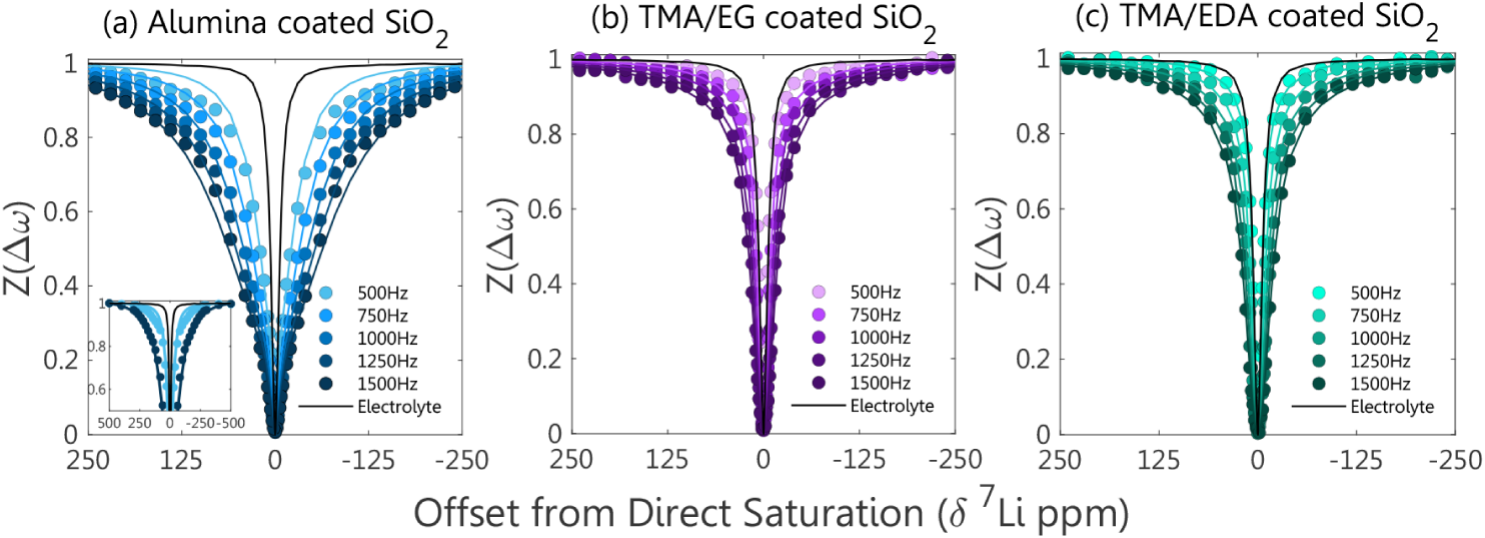
^7^Li DEST profiles (circles) for the (a) Al_2_O_3_, (b) TMA/EG, and (c) TMA/EDA coatings were acquired with a 1 s saturation pulse and varying saturation amplitudes at 298 K. The profiles were fitted (lines) using the numerical solution to the two-pool BM exchange model. The initial and final fitting parameters, as well as the parameter boundaries, are detailed in the Tables 1 and 2. The inset in (a) shows the full saturation range for the 500 and 1500 Hz saturation amplitudes.

**Table 1:** Fitting Parameters and Boundaries

Parameter	Initial Value	Fixed Value	Lower Boundary	Upper Boundary
△ωf [ppm]	0	0	-	-
*R*_1f_ [Hz]	0.32 (298 K)	0.32 (298 K)	-	-
	0.26 (313 K)	0.26 (313 K)		
	0.22 (323 K)	0.22 (323 K)		
*R*_2f_ [Hz]	3	-	0.3	20
△ω_b_ [ppm]	0	-	–2	+2
*R*_1b_ [Hz]	0.1	0.1	-	-
*R*_2b_ [kHz]	30	-	20	40
*p*_b_	0.002 (TMA/EG)	0.002 (TMA/EG)	-	-
	0.002 (TMA/EDA)	0.002 (TMA/EDA)		
	0.03 (Al_2_O_3_)	0.03 (Al_2_O_3_)		
	0.028 (TiO_2_)	0.028 (TiO_2_)		
*k*_bf_ [Hz]	100	-	0	1000

The fitted profiles described the experimental data very well, clearly following both the on- and off-resonance regions. The fits reproduce the increasing breadth of the profiles with increasing temperature, as well as the separation between the different power levels, which varies from system to system. These stem from the increase in *R*_2b_ and k_bf_ with increasing temperatures, as reflected by the final fitted parameters (Table 2, values in italic were fixed in the simulations).

**Table 2:** Final Fitted Parameters

TMA/EG coating	GOF (*R* ^2^)	*p*_b_	*R*_2b_ [kHz]	*R*_2f_ [Hz]	*k*_bf_ [Hz]
298 K	0.96	*0.002*	20 ± 3	5 ± 0.2	130 ± 10
313 K	0.97	*0.002*	23 ± 3	7 ± 0.2	150 ± 10
323 K	0.93	*0.002*	44 ± 4	9 ± 0.5	650 ± 79

TMA/EDA coating	GOF (*R* ^2^)	*p*_b_	*R*_2b_ [kHz]	*R*_2f_ [Hz]	*k*_bf_ [Hz]
298 K	0.96	*0.002*	16 ± 2	6 ± 0.2	160 ± 20
313 K	0.96	*0.002*	26 ± 3	9 ± 0.2	190 ± 20
323 K	0.96	*0.002*	38 ± 3	10 ± 0.3	370 ± 30

Al_2_O_3_ coating	GOF (*R* ^2^)	*p*_b_	*R*_2b_ [kHz]	*R*_2f_ [Hz]	*k*_bf_ [Hz]
298 K	0.87	*0.03*	6.8 ± 0.3	6 ± 1	120 ± 8

TiO_2_	GOF (*R* ^2^)	*p*_b_	*R*_2b_ [kHz]	*R*_2f_ [Hz]	*k*_bf_ [Hz]
298 K	0.86	*0.028*	5.1 ± 0.3	4.9 ± 0.9	94 ± 7
313 K	0.87	*0.028*	6.6 ± 0.3	5.0 ± 0.9	100 ± 6
323 K	0.78	*0.028*	9.0 ± 0.5	6 ± 1	109 ± 8

Fitting the DEST profiles provides us with direct insight into the Li-ion affinity of the Al_2_O_3_ and organic-aluminum hybrid coatings, in the form of the release rate of Li ions from the surface, *k*_bf_. The room-temperature exchange rates obtained for both the Al_2_O_3_ and organic-aluminum hybrid coatings are of similar value, within error, while for TiO_2_ k_bf_ was slower. For both organic-aluminum hybrid coatings and the TiO_2_ particles, *k*_bf_ increases with temperature, as expected with increased thermal energy, yet with different growth rates. *R*_2*b*_ was found to increase with temperature as well, an effect we have also observed in CEST experiments probing the exchange between Li metal and its SEI.^39^ This effect was attributed to the limitation of using a two-site model to describe a very heterogeneous system, and we expect a more complex model may describe the system more faithfully at the cost of larger uncertainties in the fitted values. In principle, the variable temperature measurements could give insight into the activation energy of Li-ion desorption. A plot of the natural logarithm of the exchange rate vs the inverse of the measurement temperature (Figure 8) displayed the expected linear dependence in the case of TiO_2_ based on the Arrhenius equation. The activation energy of the exchange process was determined from the slope of the linear fit as *E*_a_ = 4.7 ± 0.2 kJ/mol. This value is qualitatively consistent with recent molecular dynamics simulations for a process of partial desolvation.^80^ The exact structure and energetics of the bound state will vary with the chemical composition of both the surface and electrolyte, an aspect that we will explore in future work. Interestingly, the same plot was not linear for both of the organic-aluminum hybrid coatings. This deviation could be explained by enhanced dynamic properties of the organic moieties on the surface of the hybrid coatings at higher temperatures.^81,82^ Conformational changes, as well as increased mobility of the surface binding sites, could trigger a change in the exchange regime, leading to the faster exchange observed at 323 K. As organic surface moieties are considerably more flexible than inorganic surfaces, it is expected that the TiO_2_ surface would not be sensitive to this effect.

**Figure 8:**
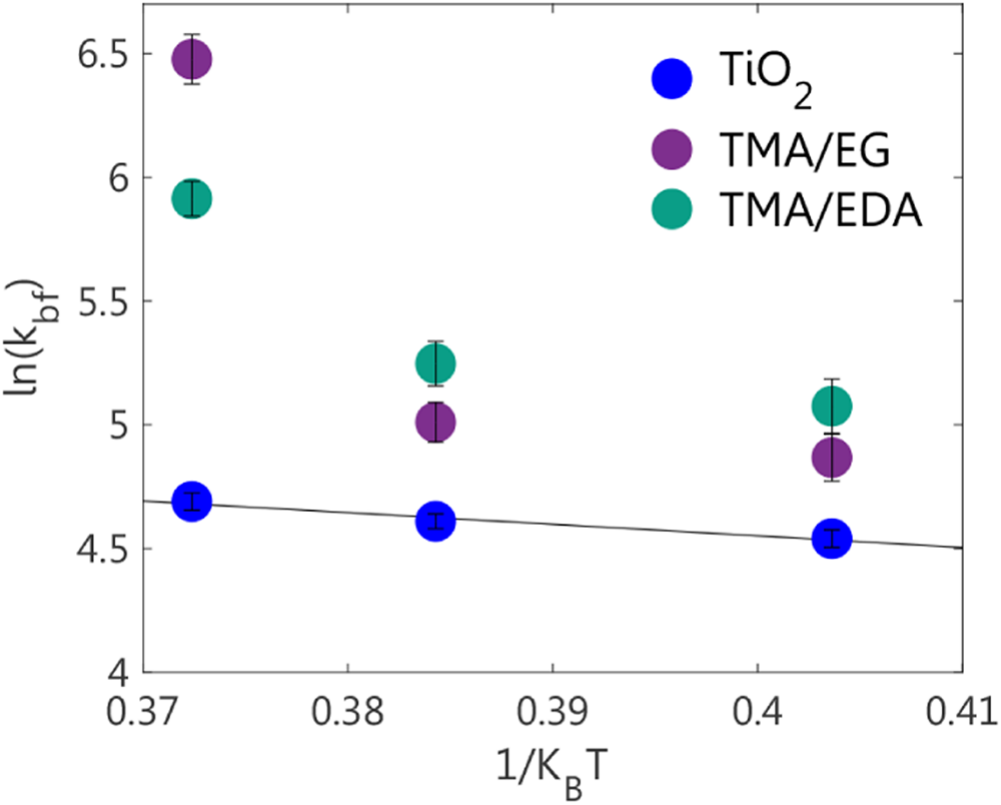
Fitted *k*bf values obtained for the two organic-aluminum hybrid coatings and the TiO_2_ particles at 298, 313, and 323 K plotted according to the Arrhenius equation. The TiO_2_ surface displayed a linear dependence, while the two organic-aluminum hybrid coatings did not.

Another possibility is that the surface adsorption of Li ions is not a simple first-order reaction. This is reasonable under the proposed model, in which the Li ion must first desolvate before binding to the surface layer. This result implies that these two processes, desolvation and binding, may occur in succession and not simultaneously, each with its own temperature dependence. We suggest that the first desolvation step is governed by the affinity of the surface toward the solvent in which the Li ion is encased. As the Li ion is known to coordinate strongly to its solvation sheath in the electrolyte solution, it would be difficult to discuss the surface affinity toward the Li ion without considering the solvent. Providing solvent accessibility to the surface and overcoming the desolvation energy barrier dictate the surface exchange rate by the strength of the Li-ion surface binding sites. Understanding the forces governing the desolvation process and its dependence on the electrolyte, as well as the surface chemistry, is currently the subject of investigation using this newly developed DEST approach.

## 4 Discussion

Applying the DEST approach to several surface chemistries, including state-of-the-art electrode coatings, enables us to evaluate their Li affinity for the first time and to compare them in the context of Li-ion transfer across the EEI. As mentioned in the introduction, Li-ion surface binding is the first step in the migration process of Li ions across the interface. To date, it has not been possible to isolate this step from migration across the EEI and charge transfer with the electrode. In principle, we expect the binding process to depend strongly on the outer chemistry of the EEI. Clearly, increasing the number of binding sites, which in our model corresponds to *p*_b_, would have a positive effect on the ion transfer properties of the EEI. In contrast, it is harder to predict what surface exchange rate would lead to the best transport, in line with the Sabatier principle. Our DEST measurements bring us closer to evaluating this by allowing us to rank the different surface chemistries by their binding ability, which should increase with *p*_b_ and have an intermediate value of *k*_bf_. These values are summarized in Figure 9 for the chemistries examined here. Our results suggest that SiO_2_ is likely binding Li ions too strongly, which would lead to increased interfacial resistance. Alumina and titania have a large number of binding sites and a high exchange rate, while the organic functionalities have a slightly higher exchange rate, yet their number of binding sites is significantly lower.

**Figure 9:**
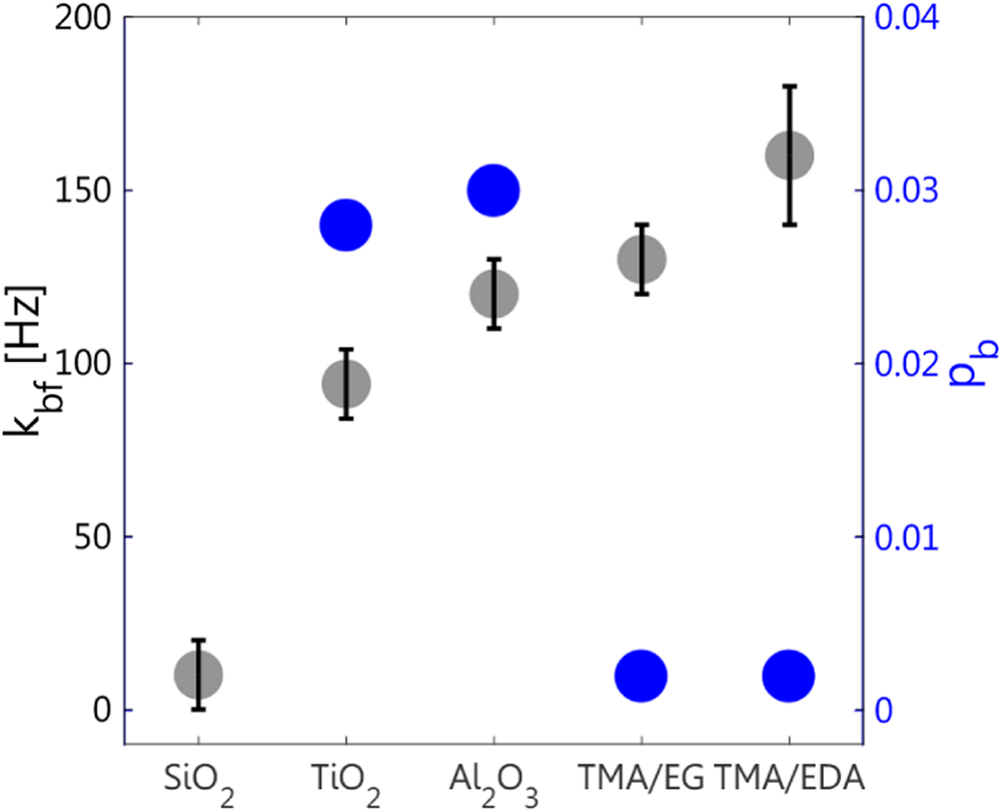
*k*_bf_ (gray) and *p*_b_ (blue) values were obtained for all the measured surface chemistries at 298 K. The values were derived from the fitted ^7^Li DEST profiles, except for SiO_2_ for which simulations were used to estimate the values.

We note that the most meaningful parameter would be the activation energy for the exchange process. In principle, this can be obtained by determining the temperature dependence of the exchange rate. Here, this approach was attempted for two types of coatings, where we found that at elevated temperatures, the DEST process is complicated by additional motional processes (for example, rotation of organic moieties) or surface reactivity (in the case of alumina). When possible, i.e., when significant exchange is occurring at room temperature, performing DEST measurements at lower temperatures may be a way to derive the activation energy. In that case, the activation energy can be compared directly to the activation energy obtained from modeling. Comparison with values obtained from EIS measurements could be used to determine whether electrolyte desolvation is a rate-limiting step in interfacial ionic charge transfer.

Considering only the exchange rates obtained from DEST, in the set of chemistries studied here, we find that inorganic coatings such as titania and alumina bind Li ions slightly better than the organic moieties we have examined. It is likely that exploring a wider range of organic functionalities would lead to organic moieties that can compete well with the electrolyte solvation sheath and outperform the inorganic surfaces. This aspect is intriguing to explore further, especially considering the structure of the native SEI, which is believed to consist of an inner inorganic layer and outer organic layer.^4,5^ The organic SEI species are known to form by decomposition of the electrolyte solvent molecules, which contain coordinating oxygen groups, while the source of the inorganic SEI species is from the anion and additional additives. The organic outer layer is considered permeable to the electrolyte, providing the medium for Li-ion transfer toward the inorganic inner layer, presumed to be the main passivating as well as ion-conducting interface. The methodology developed here offers a powerful tool to examine the role of polar organic surface species in Li binding. Furthermore, this approach may be used to design artificial SEI layers, where the presence and role of organic species are often overlooked.^26^

We did not consider interphases containing lithium as part of their composition here (such as Li_2_O, Li_2_CO_3_, LiF, and organic lithium species like poly­(ethylene oxide) and carbonate fragments; all are commonly found in the SEI of lithium anodes). Nevertheless, we foresee that the presented Li-DEST approach can also be employed for such lithiated interphases (native or artificial) in a qualitative manner, with quantification likely requiring a more complex exchange model containing several lithium pools.

## 5 Conclusions

In this work, ^7^Li DEST was applied for the first time to investigate the surface adsorption process of Li ions on electrode and electrode-coating materials. This straightforward approach allows for direct qualitative comparison of the binding properties of different surface layers, as well as a quantitative evaluation using numerical Bloch–McConnel exchange models. Utilizing model systems of monodisperse particles allowed for detailed examination of the parameters affecting the DEST measurement as well as the determination of the surface chemistry effect. Implementation of our optimized method on common electrode coatings directly attests to their Li-ion adsorption capabilities, previously inferred from convoluted electrochemical measurements. Moreover, valuable insight was gained into the Li-ion binding role of organic surface species, previously thought to provide only mechanical benefits to surface layers.^80–82^

This approach can be utilized to study the role of individual surface elements found in artificial as well as native EEIs, promoting the development of beneficial artificial coatings and electrolyte additives. Finally, applying the DEST methodology to different electrolyte systems could help elucidate the Li-ion solvation sheath and desolvation energy.

## Supplementary Material



## Notes

Additional experimental details and results: Experimental and calculated parameters for the TiO_2_ and SiO_2_ spherical particles; Quantification of available surface area from solvent ^1^H relaxation; ^7^Li DEST measurement on high surface area SiO_2_ particles; Assignment of residual TEOS signal in ^13^C ssNMR DNP-SENS measurements; Supporting experiments and calculations used to determine parameters boundaries for ^7^Li DEST fittings; Two-pool exchange model, simulations and fittings (PDF)
