# Supplementary material for: Elucidating Surface Adsorption of Lithium Ions on Electrode Materials Using 7Li Dark-State Exchange Saturation Transfer NMR Spectroscopy
Source: J Am Chem Soc. 2025 Sep 26;147(40):36277–90. doi: 10.1021/jacs.5c09603 (PMC12512186; doi:10.1021/jacs.5c09603)
Supplement: Supplementary file 1 [file ja5c09603_si_001.pdf]

Supporting Information

for

Elucidating Surface Adsorption of Lithium Ions on Electrode Materials using ^{7}Li Dark-State Exchange Saturation Transfer NMR spectroscopy

Shakked Schwartz(1), Ayan Maity(1), Vaishali Arunachalam(1), Yuval Bernard(1), Ortal Lidor-Shalev(2,3), Tehila Meshita(2,3), Liat Avram (4), Malachi Noked(2,3) and Michal Leskes(1)*

(1) Department of Molecular Chemistry and Materials Science, Weizmann Institute of Science, Rehovot, 7610000, Israel
(2) Department of Chemistry, Bar -Ilan University, Ramat Gan, 529002, Israel.
(3) Bar -Ilan Institute of Nanotechnology and Advanced Materials, Ramat Gan, 529002, Israel.
(4) Department of Chemical Research Support, Weizmann Institute of Science, Rehovot, 7610000, Israel
*michal.leskes@weizmann.ac.il

Contents

Supporting Information 1

1. Additional Experimental Details and Results 2

1.1 Experimental Parameters and Calculations 2

1.2 Surface area from solvent ^{1}H relaxation 3

1.3 Understanding Li-ion binding to SiO_2 4

1.3.1 ^{7}Li DEST on dendritic fibrous nanosilica (DFNS) 4

1.3.2 SiO_2-bound Li-ions 4

1.3.3 Comparing SiO_2 and TiO_2 surface chemistry 5

1.4 Residual TEOS in bulk SiO_2 6

1.5 Reactivity of the Al_2O_3 coating 7

1.6 Supporting experiments and calculations used to determine parameters boundaries 7

1.6.1 Estimation of the surface-bound Li-ion relaxation properties: R_{1b} and R_{2b} 8

1.6.2 Confinement effect on Li-ion free pool relaxation rates 9

1.7 Two-pool exchange model, simulations and fittings 10

1.7.1 Bloch-McConnel (BM) two-pool exchange model 10

1.7.2 Numerical simulations of ^{7}Li-DEST profiles 11

2. References 13

1. Additional Experimental Details and Results

1.1 Experimental Parameters and Calculations

A geometrical estimation of the total surface area in each sample, the number of binding sites and the theoretical p_b were calculated based on the quantity of particles and electrolyte in each sample and the density of the material. As the TiO_2 and SiO_2 particles are spherical, their volume and surface area could be calculated based on the equations for the volume and surface area of a sphere. The results of the calculations are described in the table below:

	TiO_2		SiO_2	
	**Experimental Parameters**			
Total Particle Weight (mg)	40±0.4		30±0.3	
Total Electrolyte Volume (μL)	20.0±0.2		30.0±0.3	
Particle Density (g/cm^3)	3.8[1]		2.2[1]	
	**Calculated parameters**			**Equations**
Particle Diameter (nm)	500±20	200±30	310±20	
Particle Surface Area (m^2)	$7.9 \pm 0.6 \times 10^{-13}$	$1.3 \pm 0.4 \times 10^{-13}$	$3.0 \pm 0.4 \times 10^{-13}$	$SA_{particle} = 4\pi \left(\frac{d}{2}\right)^2$
Particle Volume (cm^3)	$6.5 \pm 0.8 \times 10^{-14}$	$4 \pm 2 \times 10^{-15}$	$1.6 \pm 0.3 \times 10^{-14}$	$V_{particle} = \frac{4}{3}\pi(\frac{d}{2})^3$
Number of particles	$1.6 \pm 0.2 \times 10^{11}$	$2.5 \pm 0.8 \times 10^{12}$	$9 \pm 2 \times 10^{11}$	$\frac{V_{total}}{V_{particle}} = \frac{W_{total}/\rho}{V_{particle}}$
Total Surface Area (m^2)	$1.27 \pm 0.05 \times 10^{-1}$	$3.2 \pm 0.4 \times 10^{-1}$	$2.6 \pm 0.2 \times 10^{-1}$	$SA_{particle} \times \# particles$
Number of binding sites	$3.5 \times 10^{17+} \pm 0.2$	$9 \times 10^{17+} \pm 1$	$7.3 \pm 0.5 \times 10^{17}$	$\frac{SA_{total}}{36\ Å^2}$
Number of Li-ions	$1.20 \pm 0.08 \times 10^{19}$	$1.20 \pm 0.08 \times 10^{19}$	$1.80 \pm 0.08 \times 10^{19}$	$(C \times V)_{electrolyte} \times N_{AV}$
upper limit of p_b	0.030 ± 0.003	0.07 ± 0.01	0.041 ± 0.005	$\frac{\#binding\ sites}{\#\ Li^+}$

1.2 Surface area from solvent ^{1}H relaxation

An estimate of the Li-ion accessible surface area in the packed-particle system was obtained by correlating the solvent relaxation rate in dilute particle suspensions with the geometrical surface area of the spherical particles. Near-surface molecules are characterized by enhanced relaxation rates, and in dilute suspensions this effect is linearly correlated with the geometrical surface area[2,3]. ^{1}H Relaxation rates of dilute suspensions with varying amounts of SiO_2 (310±20 nm) particles in hexane were measured (Figure S1). Hexane was chosen as the solvent assuming it would have negligible interactions with the silica surface. Using the experimental R_1 value, the surface area of the real, packed particle system was extrapolated from the linear fit and found to be $5.6 \pm 0.6\ \frac{m^2}{g}$, while the geometrical surface area was found to be $8.6\ \pm 0.6 \frac{m^2}{g}$. The geometrical surface area was calculated as described in the previous section. The ratio of the extrapolated and calculated surface area values was designated as the 'packing factor', $f_p\ = 1.5 \pm 0.2$,which quantifies the reduction of the available surface area in the system compared to the geometrical surface area due to packing. The maximum number of binding sites was estimated from the extrapolated surface area, assuming a high density of binding site, every 36 A^2.

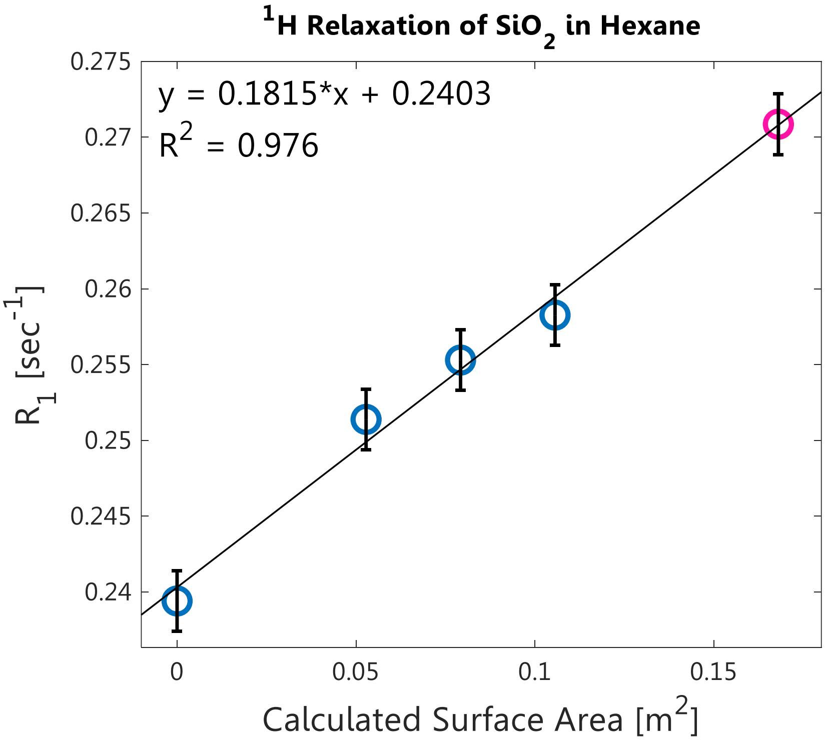

Figure S1 – ^{1}H longitudinal relaxation rate (R_1) of hexane in infinite and dilute suspensions of SiO_2 (containing 0, 6 ,9 and 12 mg SiO_2 in 30 μL hexane) as a function of the calculated surface area are plotted in blue. The linear fit was used to extrapolate the surface area of the real, packed particle system using the experimental R_1 value, plotted in pink (30 mg SiO_2 in 30 μL hexane). ^{1}H R_1 values were measured at 298 K, using the inversion recovery (IR) sequence, as detailed in the experimental section.

Accordingly, the upper limit of the bound Li-ion fraction was estimated by $\frac{\#binding\ sites}{\#\ Li^{+}}$, and found to be $p_b = 0.027 \pm 0.03$.

1.3 Understanding Li-ion binding to SiO_2

1.3.1 ^{7}Li DEST on dendritic fibrous nanosilica (DFNS)

To understand the origin of the lack of DEST effect in the case of the SiO_2 particles, additional ^{7}Li DEST measurements were conducted on dendritic fibrous nanosilica (DFNS)[4], which feature 10 times larger surface area than the spherical SiO_2 particles. Remarkably, both DEST profiles were identical, indicating the p_b was not the limiting factor in this case, but the strong binding of the Li-ions to the SiO_2 surface, resulting in ultra-slow exchange.

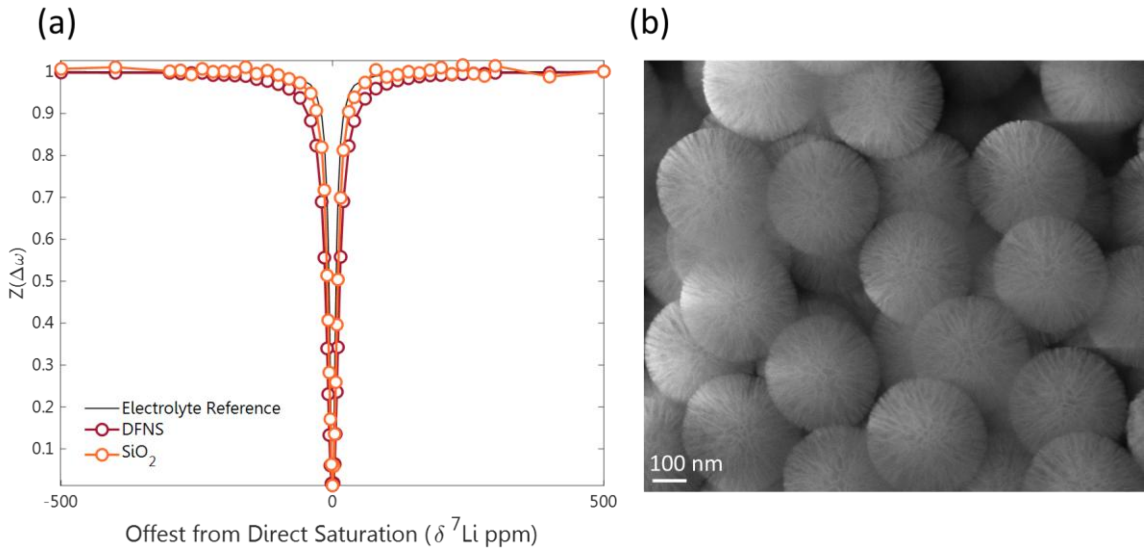

Figure S2 – (a) ^{7}Li DEST profiles of the SiO_2 and DFNS particles immersed in 1M $LiPF_6$ electrolyte at 298K, measured with a 1 kHz and 1 second saturation pulse, compared with a pure electrolyte DEST profile. (b) SEM image of the DFNS particles

1.3.2 SiO_2-bound Li-ions

Another source of the lack of broadening in the DEST profile can be due to the bound state having very long T_2 relaxation which would result in a narrow DEST profile. To test this experiments were performed on SiO_2 particles that were soaked in $LiPF_6$ electrolyte overnight, followed by washing and drying. The particles were then measured by ^{7}Li ssNMR at room temperature (298 K). We observed a very broad ^{7}Li signal centered around 0 ppm with a FWHM of approximately 50 kHz. Furthermore, in a echo measurements (here shown for solid echo with similar results obtained with Hahn echo) performed with an echo delay of 10µs the broad resonance completely disappeared, showing only a small narrow signal arising from residual electrolyte. Based on the decay of the broad signal in these echo measurements, we conclude that surface-bound Li-ions will likely have $T_2 < 20\mu s$.

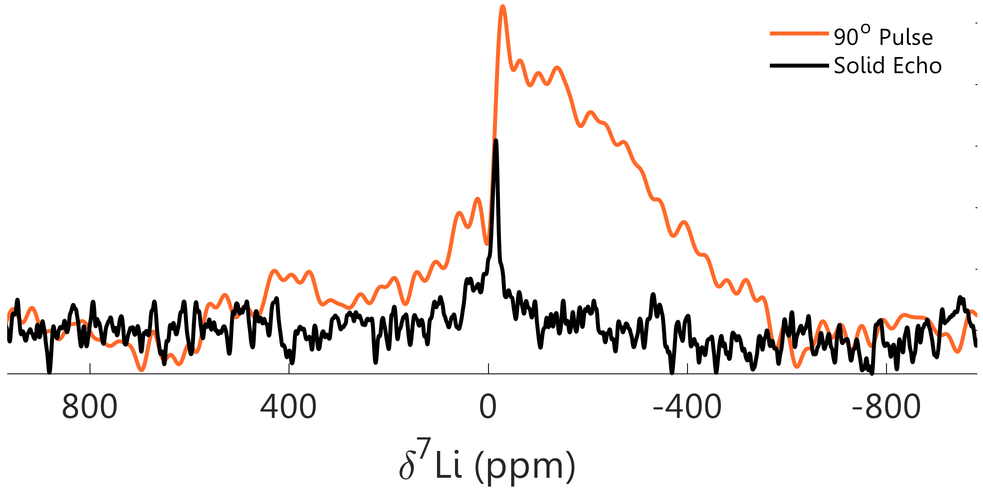

Figure S3 – ^7Li ssNMR spectra of Li ions adsorbed onto SiO_2 particles, measured with a 90° pulse (orange) and a solid echo sequence (black). The measurement was conducted without spinning, using 1 second recycle delay and 8192 scans.

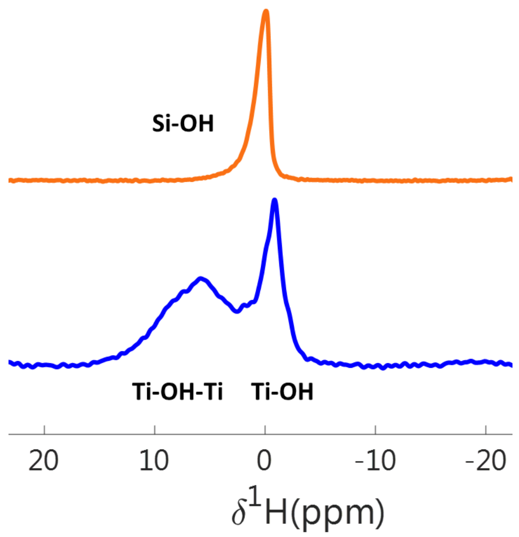

Figure S4 – ^1H ssNMR spectra of calcined SiO_2 (orange) and TiO_2 (blue) particles, measured with a hahn echo sequence, spinning at 10kHz, using a 25 and 75 second recycle delay, respectively, and 16 scans.

1.3.3 Comparing SiO_2 and TiO_2 surface chemistry

In an effort to understand the significant difference between the ^7Li DEST profiles, the surface chemistry of the SiO_2 and TiO_2 particles was examined by ^1H ssNMR. Clear differences were observed, with the TiO_2 surface exhibiting both terminal and bridging hydroxyl groups and the SiO_2 surface showing only the former.[5–8] The DEST measurement is indeed sensitive to the differences in surface chemistry, but the exact mechanism of the Li-ion interaction with the surface remains unknown. A possible mechanism of surface adsorption is Li^+-H^+ exchange with the surface, which could lead to the different exchange regimes observed for SiO_2 and TiO_2. However, as both surface contain OH group it is not trivial to predict which one would lead to tight binding. These

difference will be explored in more details in future NMR measurements before and after exposure to Li-ion electrolyte which may shed more light on this surface adsorption process.

1.4 Residual TEOS in bulk SiO_2

Both coated and uncoated SiO_2 samples displayed a ^{13}C methyl signal in ^{13}C ssNMR DNP-SENS measurements (Figure 5c). This environment was separately identified by ^{29}Si ssNMR DNP-SENS measurements (Figure S3), which displayed a signal centered around -20 ppm corresponding to SiO_2R_2 species known as a D site[9,10]. We deduced these two signals belong to partially decomposed TEOS, the silicon precursor in the synthesis. As all samples underwent high temperature calcination as well as ozone plasma treatment prior to the coating process and measurements, we suspected this signal originated from the bulk of the particle. This was confirmed by the ^{29}Si ssNMR DNP-SENS spectra, which revealed the organosilicon signal only in measurements performed with long recycle delays. In general, DNP-SENS measurements are biased towards surface species due to their proximity to the external polarizing agents, enabling them to build up hyperpolarization faster than the bulk environments[11]. Thus, the longer the build-up time required for the signal to appear, the deeper it resides below the surface. Thus, we conclude that the TEOS decomposition products, corresponding to the methyl and an alkoxy groups observed in the ^{13}C ssNMR DNP-SENS spectrum, are trapped beneath the surface of the particle and do not affect the surface exchange measurements.

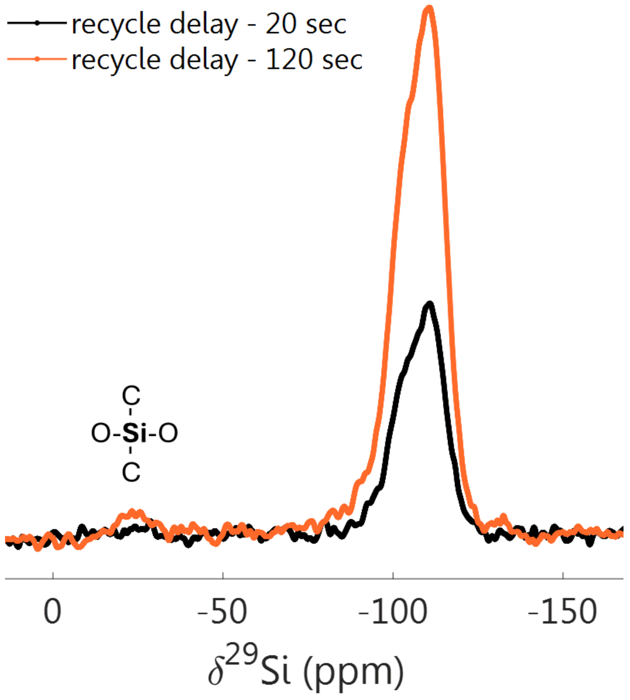

Figure S5 – Direct excitation ^{29}Si ssNMR DNP-SENS spectra of ozone-treated, pristine SiO_2 particles, using 20 seconds (black) and 120 seconds (orange) recycle delays.

1.5 Reactivity of the Al_2O_3 coating

Measurements of ^{7}Li DEST on the Al_2O_3 coated SiO_2 particles revealed that the Al_2O_3 surface was sensitive to the $LiPF_6$ electrolyte. At temperatures higher than 298 K, ^{7}Li signal loss was observed during the measurement and bubbles were formed inside the sample. At room temperature, the ^{7}Li signal remained stable, suggesting that at this temperature the surface remained unreactive.

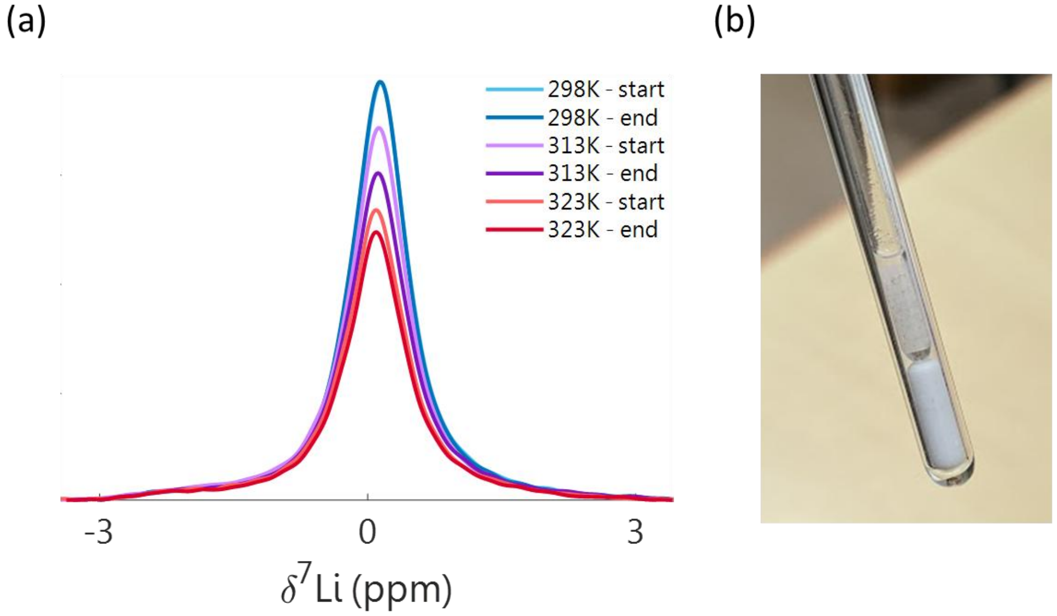

Figure S6 – (a) ^{7}Li NMR spectra of Al_2O_3 coated SiO_2 particles immersed in 1M $LiPF_6$ electrolyte before and after each DEST measurement, which were measured at different temperatures. (b) The sample at the end of the 323K measurement, displaying bubbles formed during the experiment, indicating a chemical reaction.

1.6 Supporting experiments and calculations used to determine parameters boundaries

The DEST process and resulting measured profile are affected by several parameters, some of which provide insight into the chemistry and dynamics of the system directly and some are a result of the magnetic resonance properties of the involved species. As we aim to obtain insight into the dynamics of the system, we strived to remove as many uncertainties as possible into the system properties as well as determine realistic boundaries for the various parameters.

1.6.1 Estimation of the surface-bound Li-ion relaxation properties: R_{1b} and R_{2b}

To better understand the behavior of the surface-bound Li-ion population and to obtain accurate boundaries for our simulations and fitting model we attempted to isolate this environment using static ssNMR measurements. Al_2O_3 coated SiO_2 particles were soaked in $LiPF_6$ electrolyte overnight, followed by washing and drying. The particles were then characterized by ^7Li ssNMR at room temperature (298 K). The measurement displayed a single peak centered around 0 ppm, made up of a

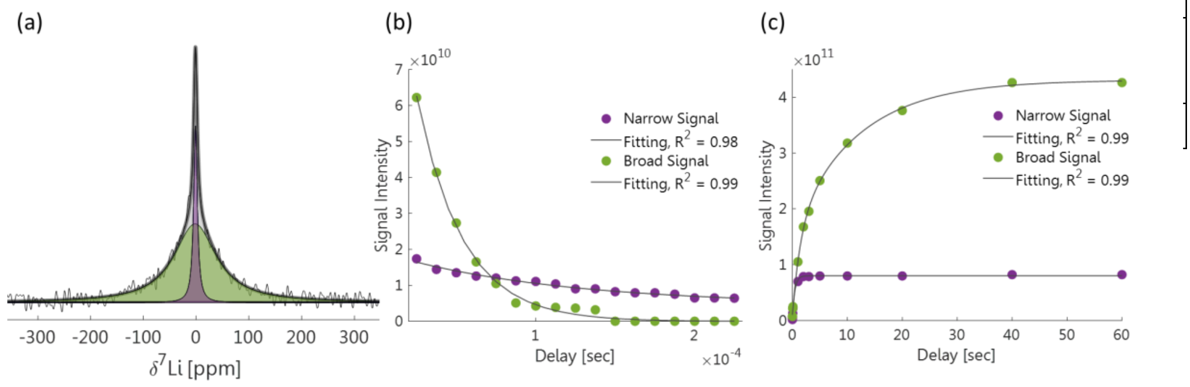

Figure S7 – (a) Deconvoluted ^7Li ssNMR spectrum of Li ions adsorbed onto Al_2O_3 coated SiO_2 particles. The measurement was conducted without spinning, using 30 seconds recycle delay. (b) T_2 Hahn echo and (c) T_1 saturation recovery measurements of the deconvoluted signal.

broad and a narrow component (Figure S4a). We deduced the broad component represents the surface adsorbed Li-ions while the narrow component belongs to more mobile Li-ions, perhaps still coordinated to residual solvent molecules, yet still not as mobile as the free Li-ion population in the sample measured by ^7Li DEST (see table). In relaxation measurements conducted on the broad signal component we found very different timescales for T_1 and T_2 (Figure S4b-c). The T_2 of the surface bound Li-ions, measured by Hanh echo, was found to be $\sim 30\ \mu sec$,corresponding to a relaxation rate $R_{2,b}$ of 30 kHz. T_1 measurement by saturation recovery displayed two components, which could be due to the heterogeneity of the surface and Li-ion binding modes, as well as quadrupolar effects of ^7Li. The dominant T_1 value was $\sim 12\ sec$, corresponding to a relaxation rate $R_{1,b}$ of 0.08 Hz.

In comparison, the relaxation rates of the bulk electrolyte were very similar in magnitude, with an $R_{1,f}$ of 0.3 Hz and an $R_{2,f}$ of 0.4 Hz. These values are in agreement with the dependance of R_1 and R_2 on the correlation time in which $R_{1A} \approx R_{1B}$ and $R_{2A} \ll R_{2B}$ when the states A and B differ significantly in their molecular size[12]. In our case, states A and B refer to the free and bound Li-ion populations, respectively. This measurement represents an overestimation of the bound state which was mostly removed of solvent (unlike the bound state in the wet sample measured in DEST). As such, these relaxation rates were used as the upper boundaries for the simulations and as initial guesses in the fitting performed on the room temperature DEST profiles.

1.6.2 Confinement effect on Li-ion free pool relaxation rates

Although the free population is visible and measurable, the observed Li^{+}_{free} relaxation rates are likely affected by the surface exchange process in the confined inter-particle environment. As we are interested in the relaxation rates in the absence of exchange, we estimated the confinement effect on the relaxation rates of the free population following the method described by Godefroy et al.[13,14]

Liquids confined in porous structures have relaxation contributions from the bulk and near-surface populations. Under the condition of biphasic fast exchange, which allows every molecule in the pore to briefly probe the surface, coupled Bloch equations describing the bulk and surface interactions have been solved to give the following relaxation equation[13,14]:

$$R_{1,2\,confined} = R_{1,2bulk} + \frac{2\alpha\lambda}{d_{pore}} R_{1,2\,surf} + \frac{8\alpha D}{d_{pore}^2}$$

d_{pore} represents the model pore diameter, $\alpha = 1, 2\ or\ 3$ is the shape factor for planar, cylindrical, and spherical pore geometries, respectively, and λ is a representative distance beyond which the effects of the relaxation disappear. D is the translational self-diffusion coefficient of the liquid within the pore. $R_{1,2bulk}$ and $R_{1,2surf}$ are the longitudinal and transverse relaxation rates of the bulk and surface populations, respectively, while $R_{1,2\,confined}$ are the effective relaxation rates of the confined liquid in the porous structure. The biphasic fast exchange condition implies that at steady state the surface and bulk population are exchangeable to an equal degree. The equation clearly describes the bulk and surface relaxation processes occurring in parallel, so determining the significant relaxation process will allow us to disregard the second one. As the particles in our system are spherical, by geometrical estimation $d_{pore} \cong d_{particle}$, and $\alpha = 3$ was chosen to reflect the pore dimensionality. $R_{1,2_{surf}}$ were estimated by ^{7}Li ssNMR measurements, described in the previous paragraph, and λ was estimated to be 1 nm . D was measured using ^{7}Li PFG diffusion NMR and found to be in the order of $10^{-6}\ \frac{cm^2}{sec}$ In our case $\frac{4D}{d_{pore}} \gg \lambda\, R_{1,2\,surf}$, meaning the relaxation is surface limited and the equation is reduced to:

$$R_{1,2confined} = R_{1,2bulk} + \frac{2\alpha\lambda}{d_{pore}} R_{1,2_{surf}}$$

Calculating the confined relaxation rates we found the longitudinal rate to be largely unchanged from its bulk value, 0.32 Hz, while the transverse rate increased by two orders of magnitude, from 0.3 Hz to 900 Hz. These estimates for the relaxation rates of the confined Li^{+}_{free} again represent the maximal possible values, as they were calculated using the $R_{1,b}$ and $R_{2,b}$values described above, and assuming the tightest possible particle packing. We estimated the maximum reasonable $p_b = 0.027 \pm 0.03$ for the 310 nm sized SiO_2 particles, based on geometrical considerations and the packing factor which relates the geometrical surface area and the accessible surface area in the packed-particle system. All

these estimated values were defined as the upper boundaries for the simulations and fittings performed in section 4.2.1 in the main text and section 1.6.2 in the supporting information, while the parameters measured for the free ("bulk") electrolyte were used for the lower boundaries.

1.7 Two-pool exchange model, simulations and fittings

1.7.1 Bloch-McConnel (BM) two-pool exchange model

The six BM equations describing the time- dependence of the magnetization in the two-pool model are expressed as[15]:

$$\frac{d\vec{M}}{dt} = A\vec{M} + \vec{C}$$

With the magnetization vector $\vec{M}$ (given in the rotating frame x, y, z):

$$\vec{M} = (M_{f,x}, M_{f,y}, M_{f,z}, M_{b,x}, M_{b,y}, M_{b,z})$$

, the matrix A:

$$A = \begin{bmatrix} \boldsymbol{L}_f - p_b\boldsymbol{K} & \boldsymbol{K} \\ p_b\boldsymbol{K} & \boldsymbol{L}_b - \boldsymbol{K} \end{bmatrix}$$

$$\boldsymbol{L_i} = \begin{pmatrix} -R_{2_i} & -\Delta\omega_i & 0 \\ \Delta\omega_i & -R_{2_i} & \omega_1 \\ 0 & -\omega_1 & -R_{1_i} \end{pmatrix} \quad ; \quad \boldsymbol{K} = \begin{pmatrix} k_{bf} & 0 & 0 \\ 0 & k_{bf} & 0 \\ 0 & 0 & k_{bf} \end{pmatrix}$$

And the constant vector $\vec{C}$ **:**

$$\vec{C} = \begin{pmatrix} 0 & 0 & R_{1_f}M_{0_f} & 0 & 0 & R_{1_b}M_{0_b} \end{pmatrix}^T$$

With $i = f\,, b$ for the free and bound species, $\omega_1 = \gamma B_1$, where γ is the gyromagnetic ratio of lithium and B_1 the saturation power, $\Delta\omega$ the saturation frequency, and the products of the longitudinal relaxation and the thermal equilibrium magnetizations $R_{1_i}M_{0_i}$ of the two populations under a static magnetic field $\overrightarrow{B_0} = (0 \quad 0 \quad B_0)$.

1.7.2 Numerical simulations of 7Li-DEST profiles

Numerical simulations of 7Li-DEST profiles using the two-pool exchange model with magnetization parameters within the proposed boundaries revealed that in addition to the exchange rate, $R_{2,b}$ and p_b have the largest influence on the results, both inducing broadening of the DEST profile. Varying $R_{1,b}$ and $R_{1,f}$ throughout the entire parameter range did not produce a significant change in the profiles, while increasing $R_{2,f}$ introduced broadening only of the direct saturation region, $Z(\Delta\omega \cong 0)$. We note that in our simulations we do not consider the effect of heterogeneity and anisotropic interactions on the properties of the bound state. These would result in broadening of the DEST profile as it reflects an average of exchange with all possible bound state environments. However, this choice is justified by our experiments on dry particles with adsorbed Li species (Figure S4) which indicate that the main source of broadening for the bound state resonance is the short transverse relaxation time (of the order of 10s of μs).

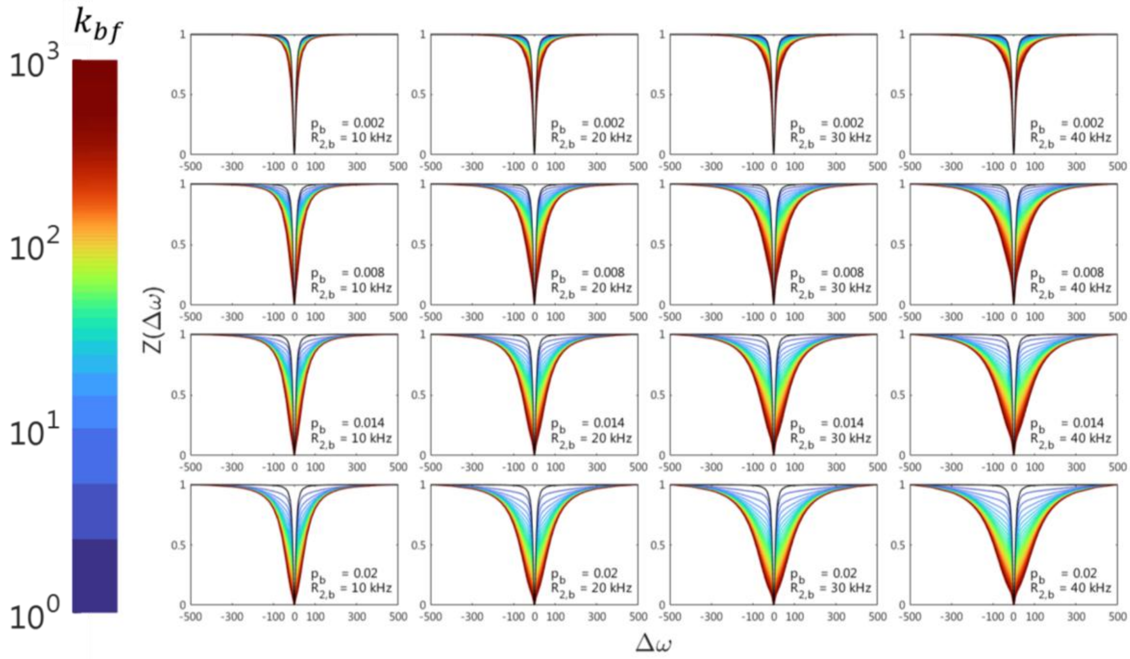

Figure S8. 7Li DEST numerical simulations based on the two-pool BM exchange model comparing the influence of $R_{2,b}$ (columns) and p_b (rows) on the resulting profiles at varying exchange rates, represented by the color gradient, and a reference profile simulated without exchange in black. The other relaxation parameters $R_{1,b}$,$R_{1,f}$ and $R_{2,f}$ were fixed at 0.1, 0.3 and 3 Hz. The chemical shifts of the bound and free states $\Delta\omega_f$ and $\Delta\omega_b$ were both set to 0 ppm.

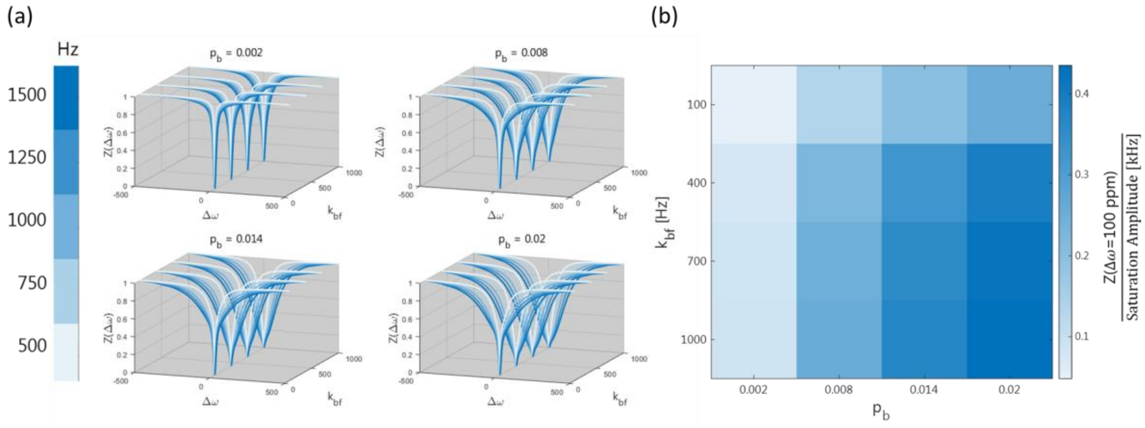

Figure S9 – (a) Simulations of [7]Li DEST profiles plotted between $\Delta\omega = \pm 500\, ppm$ conducted with k_{bf} ranging from 100 to 1000 Hz and p_b ranging from 0.002 to 0.02. The color scale represents the amplitude of the saturation pulse used in the simulation, which was kept at a length of one second. To quantify the DEST power dependency, $z(\Delta\omega) = 100\, ppm$ was plotted as a function of the saturation power, resulting in a linear fit with the slope: $\frac{\text{Z}(\Delta\omega\text{=100 ppm})}{\text{Saturation Amplitude [kHz]}}$. (b) The slope is displayed as a function of both k_{bf} and p_b, showcasing the stronger dependency of the DEST effect on the bound population vs the exchange rate.

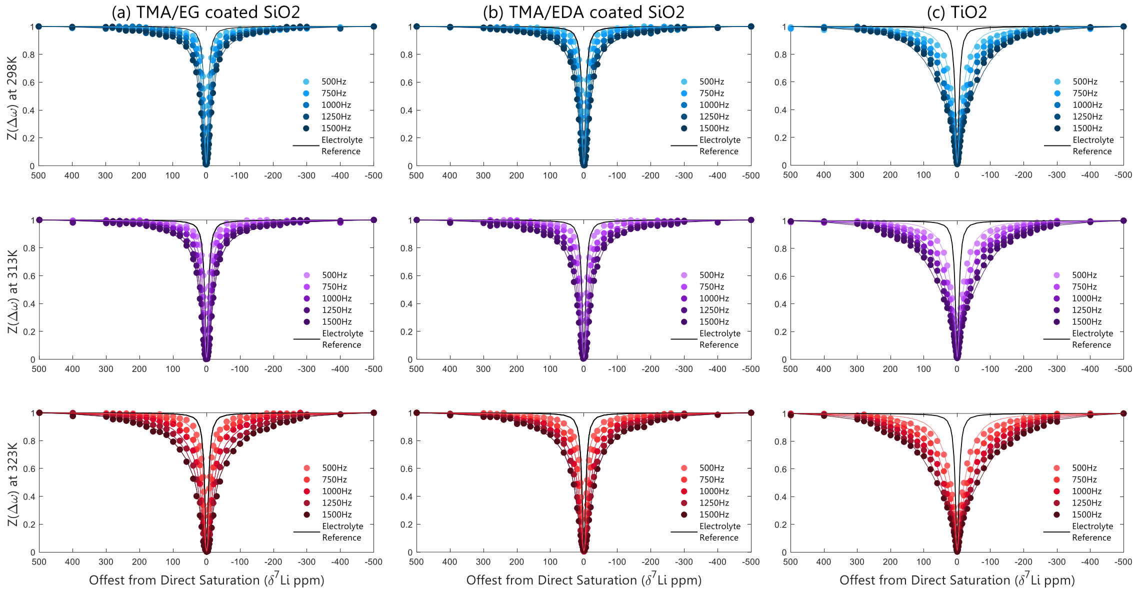

Figure S10 - [7]Li DEST profiles (circles) for (a) SiO_2@TMA/EG, (b) SiO_2@TMA/EDA and (c) TiO_2 acquired with a one second saturation pulse and varying saturation amplitudes at 298 K, 313 K and 323 K. The profiles were fitted (lines) using the numerical solution to the two-pool BM exchange model. The initial and final fitting parameters as well as the parameter boundaries are detailed in the tables in the main text.

2. References

1. Lide, D. R. *CRC Handbook of Chemistry and Physics*. vol. 85 (CRC press, 2004).

2. Davis, P. J., Gallegos, D. P. & Smith, D. M. *Rapid Surface Area Determination via NMR Spin-Lattice Relaxation Measurements*. *Powder Technology* vol. 53 (1987).

3. Cooper, C. L., Cosgrove, T., van Duijneveldt, J. S., Murray, M. & Prescott, S. W. The use of solvent relaxation NMR to study colloidal suspensions. *Soft Matter* **9**, 7211–7228 (2013).

4. Maity, A. & Polshettiwar, V. Dendritic fibrous nanosilica for catalysis, energy harvesting, carbon dioxide mitigation, drug delivery, and sensing. *ChemSusChem* **10**, 3866–3913 (2017).

5. Cracker, M. *et al.* *'H NMR Spectroscopy of Titania Chemical Shift Assignments for Hydroxy Groups in Crystalline and Amorphous Forms of Ti02*. *J. Chem. SOC., Faraday Trans* (1996).

6. Sindorf, D. W. *et al.* *) Hunger, Chromatog*. *J. Am. Chem. Soc* vol. 110 https://pubs.acs.org/sharingguidelines (1988).

7. Trébosc, J., Wiench, J. W., Huh, S., Lin, V. S. Y. & Pruski, M. Solid-state MMR study of MCM-41-type mesoporous silica nanoparticles. *J Am Chem Soc* **127**, 3057–3068 (2005).

8. Singh, R. *et al.* Probing the Interfaces in Nanosilica-Supported TiO2 Photocatalysts by Solid-State NMR and In Situ FTIR. *ChemNanoMat* **4**, 1231–1239 (2018).

9. Harris, R. K. & Kimber, B. J. 29Si NMR as a Tool for Studying Silicones. *Appl Spectrosc Rev* **10**, 117–137 (1975).

10. Williams, E. A. & Cargioli, J. D. Silicon-29 NMR Spectroscopy. in *Annual Reports on NMR Spectroscopy* (ed. Webb, G. A.) vol. 9 221–318 (Academic Press, 1979).

11. Moroz, I. B. & Leskes, M. Dynamic Nuclear Polarization Solid-State NMR Spectroscopy for Materials Research. (2022) doi:10.1146/annurev-matsci-081720.

12. An, Y., Sedinkin, S. L. & Venditti, V. Solution NMR methods for structural and thermodynamic investigation of nanoparticle adsorption equilibria. *Nanoscale Adv* **4**, 2583–2607 (2022).

13. Godefroy, S., Korb, J.-P., Fleury, M. & Bryant, R. G. Surface nuclear magnetic relaxation and dynamics of water and oil in macroporous media. *Phys Rev E* **64**, 021605 (2001).

14. Korb, J.-P. Multiscale nuclear magnetic relaxation dispersion of complex liquids in bulk and confinement. *Prog Nucl Magn Reson Spectrosc* **104**, 12–55 (2018).

15. Zaiss, M. & Bachert, P. Exchange-dependent relaxation in the rotating frame for slow and intermediate exchange–modeling off-resonant spin-lock and chemical exchange saturation transfer. *NMR Biomed* **26**, 507–518 (2013).
